# Genetic Influence of the Brain on Muscle Structure: A Mendelian Randomization Study of Sarcopenia

**DOI:** 10.1002/jcsm.13647

**Published:** 2024-11-13

**Authors:** Ting Lei, Zichao Jiang, Jiahao Wang, Jiangyu Nan, Long Hua, Zewu Zhu, Yihe Hu

**Affiliations:** ^1^ Department of Orthopedic Surgery, The First Affiliated Hospital, College of Medicine Zhejiang University Hangzhou China; ^2^ Department of Orthopedic Surgery, Hunan Engineering Research Center of Biomedical Metal and Ceramic Implants, National Clinical Research Center for Geriatric Disorders, Xiangya Hospital Central South University Changsha China; ^3^ The School of Medicine Nankai University Tianjin China; ^4^ Department of Orthopedic, The First Affiliated Hospital, Key Laboratory of High Incidence Disease Research in Xinjiang, Ministry of Education Xinjiang Medical University Urumqi China; ^5^ Department of Urology, National Clinical Research Center for Geriatric Disorders, Xiangya Hospital Central South University Changsha China

**Keywords:** brain, cross‐organ regulation, Mendelian randomization, muscle, sarcopenia

## Abstract

**Background:**

The association between brain and sarcopenia has not been clarified. We aim to investigate the causal association between brain structure, function, gene expression and sarcopenia‐related traits.

**Methods:**

All participants were Europeans. GWAS data of Brain Imaging Data Structure (BIDs) was from the UK Biobank. Gene expression in 13 brain regions was acquired from the GTEx Consortium. The sarcopenia‐related traits, including appendicular lean mass (ALM), whole body lean mass (WBLM), grip strength and sarcopenia diagnosed by the European Working Group on Sarcopenia in Older People (EWGSOP) or Foundation for the National Institutes of Health (FNIH), were from the IEU website. The inverse variance weighted (IVW), MR Egger, weighted median, weighted mode and Wald ratio methods were used for a two‐sample Mendelian randomization (MR) analysis between brain structures and sarcopenia‐related traits. The summary‐data‐based MR (SMR) was used to investigate brain genes causally influencing sarcopenia. We calculated the *F*‐value, odds ratio with 95% CI and *p*‐value. For the sensitivity analysis, the heterogeneity *I* ^2^ statistic, Cochrane's *Q* test, Egger's intercept test, MR‐PRESSO and the leave‐one‐out sensitivity test were used. Kyoto Encyclopedia of Genes and Genomes (KEGG) pathway, protein–protein interaction (PPI), transcription factor interaction prediction and miRNA interaction prediction analysis were used to reveal potential signalling pathways and mechanisms.

**Results:**

We included 3144 imaging phenotypes from 8428 participants in BIDs data. One hundred forty‐one BIDs were identified to causally influence ALM, 160 BIDs showed significant causal effect on the WBLM, and 86 BIDs showed significant causal effect on grip strength. There were 48 or 32 BIDs causally associated with sarcopenia diagnosed by the EWGSOP or FNIH criteria respectively. After FDR correction, there were 35 BIDs showing causal effect on the ALM, 28 BIDs showing causal effect on the WBLM and 7 BIDs showing causal effect on grip strength (*p* < 0.05). Twelve to forty‐eight genes in different brain regions showed causal effect on all the five sarcopenia‐related traits. MMP24‐AS1, HLA‐DRB6, HLA‐DQA2, DDX42, BAG6, NUSAP1, LINC00940, NME1‐NME2 and AS3MT in the amygdala region showed detrimental effect on all the five sarcopenia‐related traits, whereas HLA‐DRB1, HLA‐DQB1‐AS1 and C6ORF3 showed protective effect (*p* < 0.05). The gene enrichment analysis indicated these screened genes was mainly enriched in immune‐related signalling.

**Conclusion:**

We discovered the causal effect of BIDs and brain gene expression on sarcopenia. The positive genes were mainly enriched in immune‐related signalling, suggesting an immune‐based cross‐organ regulation mechanism of brain–muscle axis.

## 1 Introduction

Sarcopenia is a condition of muscle atrophy characterized with progressive loss of muscle mass and strength. It has two types, the primary one and the secondary one. Primary sarcopenia is believed to result from the natural ageing process. It could be influenced by many factors, such as hormonal levels, lack of physical activity and changes in muscle metabolism [1]. Secondary sarcopenia is usually caused by some chronic diseases, such as cancer, heart failure or chronic obstructive pulmonary disease (COPD) [2, 3, 4]. It could happen at any age for those with comorbidities. However, the specific mechanisms of both primary and secondary sarcopenia remain largely unknown. According to previous studies, sarcopenia would result in increased adverse outcomes, including but not limited to fracture, mortality, hospitalization and mental health [5]. To treat sarcopenia, exercise and nutrition therapies have been proven to improve muscle mass and muscle strength; however, the effects are limited [1, 6]. Exercise is also not suitable for those without exercising abilities. No medications are available for sarcopenia at present, either. Therefore, it would be of great significance to explore the mechanism behind the development of sarcopenia to look for new effective therapy targets.

Recently, there has been increasing evidence demonstrating that muscle interacted with the brain. For example, it was reported that cytokines secreted by muscle tissue may play a regulatory role in brain activities, such as learning, memory and mood via the gut mediation or directly through the brain [7]. And researchers found that regular exercise restored normal hypothalamic function in obese patients, implying a possible relationship between the muscle and the hypothalamus [8]. In addition, a long‐term cohort study have found that sarcopenia would accelerate the volume loss of brain region, especially in the parietal lobe [9]. These results imply that the altered homeostasis of muscle could cause effects on the function and volume of brain. Whether the brain could affect the incidence of sarcopenia is little known. One research based on NHANES and Mendelian randomization (MR) analysis found that cognitive performance showed positive causal effects on walking speed and ALM of participants, meaning that brain functions could influence muscle mass and performance from genetic aspects [10]. However, no studies have provided conclusive evidence of a causal relationship between brain structure functions and muscle. It would benefit to look for new targets for sarcopenia diagnosis and treatment to investigate the relationship between brain structures, functions and sarcopenia.

Due to the limitation of ethical principle and high cost, it was difficult to perform clinical studies or epidemiological studies to investigate whether altered brain structure or function affect the function of muscle. MR is an epidemiological strategy that uses measurable genetic variants to investigate the causal effect of exposure on outcomes, which could solve these shortcomings of traditional clinical investigations [11]. This method follows Mendel's first and second laws of genetic inheritance: the law of separation and the law of independent classification [11]. Hence, reverse causation and confounding are less likely to occur with this method than with conventional observational research [12]. However, no MR‐based studies have discussed the causal relationship between brain structures, functions and sarcopenia before. In this study, we aimed to use MR analysis to explore whether the changes of brain structure and gene expression in brain regions would cause any effect on sarcopenia‐related traits, to provide better understandings for sarcopenia diagnosis and treatment.

## 2 Data and Methods

### 2.1 Study Design

**FIGURE 1:**
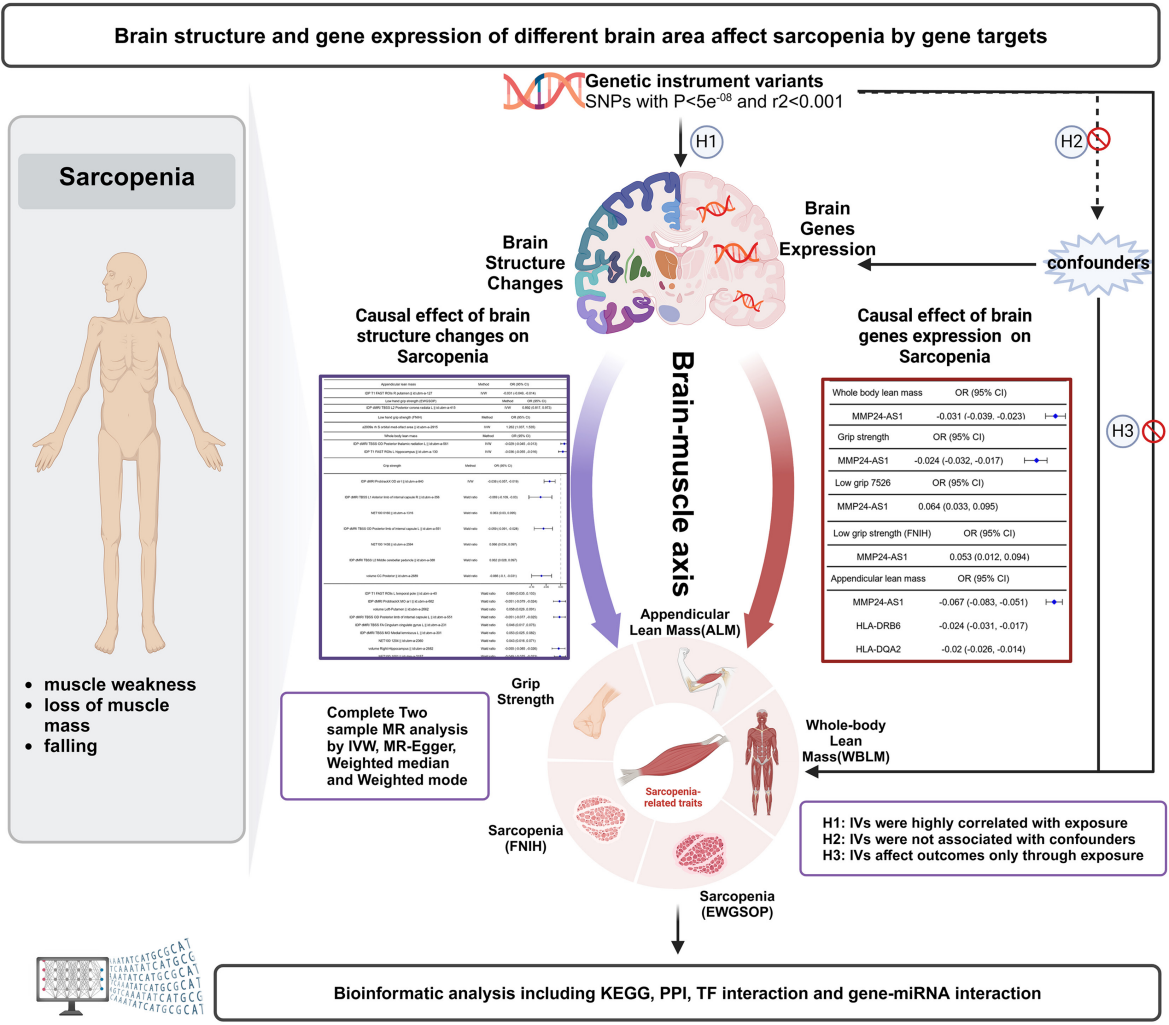
Schematic diagram of brain structure, function and gene expression affecting muscle development.

Figure 1 illustrates how we employed the MR method to explore the causal relationship between brain structure, gene expression across various brain regions, and traits associated with sarcopenia (Created in BioRender. Zhou, Y. (2024) BioRender.com/j68x890). The initial phase of our research focused on determining the causal impact of brain structure on sarcopenia‐related traits, which include appendicular lean mass (ALM), whole body lean mass (WBLM), grip strength and sarcopenia, ICD‐10‐CM (M62.84), as defined by the criteria of the European Working Group on Sarcopenia in Older People (EWGSOP) or the Foundation for the National Institutes of Health (FNIH). Subsequently, we analysed gene expression in different brain regions by incorporating 13 GWAS summary datasets that catalogued gene expression across these areas. To serve as proxies for brain structure and gene expression in these diverse regions, we selected genetic instrumental variables (IVs). Additionally, we identified single nucleotide polymorphisms (SNPs) situated within a 100 kb radius of the target genes that were significantly linked to the gene expression in various brain regions.

### 2.2 Source of GWAS Summary Dataset

The exposure data used in this study included the brain structure traits and gene expression of different brain regions. The GWAS summary data of brain structure traits was acquired from the IEU open GWAS project (https://gwas.mrcieu.ac.uk/datasets/). The brain imaging data measured through the magnetic resonance imaging (MRI) method composed of 3144 structural and functional brain imaging phenotypes, which was derived from 8428 participants from the UK Biobank (PMID: 30305740). The GWAS summary data of the gene expression in 13 brain regions were obtained through the GTEx Consortium (https://gtexportal.org/home/datasets). The 13 brain regions included brain amygdala (B1), brain anterior cingulate cortex BA24 (B2), brain caudate basal ganglia (B3), brain cerebellar hemisphere (B4), brain cerebellum (B5), brain cortex (B6), brain frontal cortex BA9 (B7), brain hippocampus (B8), brain hypothalamus (B9), brain nucleus accumbens basal ganglia (B10), brain putamen basal ganglia (B11), brain spinal cord cervical c‐1 (B12) and brain substantia nigra (B13). The primary demographic of exposure dataset consisted of Europeans.

The outcome data used in this study comprised five sarcopenia‐related traits, including ALM, WBLM, grip strength and sarcopenia diagnosed with different criteria. Additionally, traits associated with sarcopenia were gathered from the IEU website, categorized into five areas: participants with low hand grip strength as per the 2010 EWGSOP criteria (48 596 cases vs. 207 927 controls), low hand grip strength as determined by the criteria of the FNIH (20 335 cases and 236 188 controls), grip strength (sample size = 461 026), ALM (sample size = 450 243) and WBLM (sample size = 454 850). According to EWGSOP standards, males with grip strength below 30 kg or females below 20 kg were classified as sarcopenia. Following FNIH standards, males under 26 kg or females under 16 kg of grip strength were similarly classified as sarcopenia. A summary of the sarcopenia‐related datasets included is presented in Table S1.

### 2.3 SNP Selection and Two‐Sample MR Analysis

We implemented several methods, including the inverse variance weighted (IVW) method, MR Egger method, weighted median method and weighted mode method, to investigate the potential causal relationships between brain structure, traits of 13 brain regions, and sarcopenia (characterized by low grip strength, ALM and WBLM). For the MR analysis to be valid, it was crucial to adhere to three key criteria: (1) The SNPs should influence only the exposure traits, such as brain structure and the 13 brain region traits; (2) the SNPs should not be associated with confounding factors affecting the exposure traits; and (3) the SNPs should influence the outcome traits solely through their effect on the exposure traits. To ensure the selection of relevant SNPs, we applied a threshold of *p* ≤ 5e‐8 and an *r* ^2^ = 0.001, employing the linkage disequilibrium (LD) clustering algorithm to eliminate SNPs with potential LD issues, thereby mitigating complex linkage imbalance effects. Additionally, we set the gene windows for SNPs at 100 kb to enhance the precision of the MR analysis. To address the issue of pleiotropy, which could skew the results, we removed any SNP associated with the exposure if there were five or more such SNPs. Furthermore, we calculated the *F*‐value to assess the effectiveness of the selected SNPs in explaining the variance between brain structure, the traits of the 13 brain regions and sarcopenia, along with its associated traits. Upon finalizing SNP selection, we proceeded with the MR analysis to explore the association between brain structure, the 13 brain region traits and sarcopenia. For instances where only a single SNP was applicable, we employed the Wald ratio method. A fixed effects model was used when there were between one and three SNPs, whereas a random effects model was applied for cases with three or more SNPs. Upon completing the MR analysis, we derived the odds ratio (OR) along with the 95% confidence interval (CI) for each association. Additionally, the significance of the results was determined by the *p*‐value, with *p* < 0.05 indicating a statistically significant difference.

### 2.4 SMR Methods and Bioinformatic Analysis of Gene Expression of Different Brain Regions on Sarcopenia

We utilized the summary‐data‐based Mendelian randomization (SMR) approach to identify genes expressed in 13 different brain regions. For gene expression proxies, we extracted available expression quantitative trait loci (eQTLs) from different brain regions, obtaining the eQTLs data from the GTEx Consortium website (https://gtexportal.org/home/datasets). We then conducted SMR analysis to assess the causal impact of these genes on sarcopenia, setting the eQTLs parameters to a minor allele frequency (MAF) greater than 1% and a significance threshold of *p* < 5e‐08, including only *cis*‐eQTLs in our study. Subsequently, by intersecting genes with significant expression differences in various brain tissue areas related to sarcopenia and its associated traits, we identified target genes commonly expressed across different regions. We then conducted bioinformatics analyses on these genes, including Kyoto Encyclopedia of Genes and Genomes (KEGG) pathway analysis, protein–protein interaction (PPI) analysis, transcription factor interaction prediction analysis and miRNA interaction prediction analysis. For these analyses, we used the Network Analyst website (https://www.networkanalyst.ca/) [13]. KEGG is a comprehensive database for understanding the high‐level functions and utilities of biological systems at the molecular level, particularly valuable for analysing large‐scale molecular datasets generated by genome sequencing and other high‐throughput experiments [14]. Through KEGG analysis, we evaluated the potential signalling pathways between genes expressed in the brain and sarcopenia. For PPI analysis, we referred to the STRING interactome database, setting the confidence score between medium (400) and high (1000), with a cut‐off value of 900 and including experimental evidence. Transcription factor and gene target data were derived from ENCODE ChIP‐seq data, setting peak intensity signal < 500 and the predicted regulatory potential score < 1 as cut‐off values using the BETA Minus algorithm to predict target transcription factors. Lastly, gene‐miRNA interactions were predicted using the miRTarBase database, which validates miRNA–gene interactions through comprehensive experimental evidence. These analyses provide deeper insights into molecular interactions, laying the groundwork for future validation studies.

### 2.5 Sensitivity Analysis

To evaluate the effectiveness and consistency of the MR analysis in linking exposures to outcomes, we employed several statistical tests and methods, including the heterogeneity *I* ^2^ statistic, Cochrane's *Q* test, Egger's intercept test, MR‐PRESSO and the leave‐one‐out sensitivity test. The heterogeneity *I* ^2^ statistic and Cochrane's *Q* test were utilized to measure the variability in the results, with a *p*‐value < 0.05 indicating significant heterogeneity across different populations, which is quantitatively described by the *I* ^2^ statistic. The Egger's intercept test was applied to detect potential pleiotropy in the MR analysis outcomes. All MR analyses were performed using R software and its associated packages.

### 2.6 Ethical Statement

The data for our study were sourced from publicly available GWAS summary databases, as published on PubMed. The ethical considerations for these data were managed by the originating institutions, which obtained informed consent from all participants. Given the public nature and prior ethical approval of these datasets, our study proceeded without requiring further ethical clearance.

## 3 Results

### 3.1 The Causal Effect of Brain Structure on Sarcopenia‐Related Traits

**FIGURE 2:**
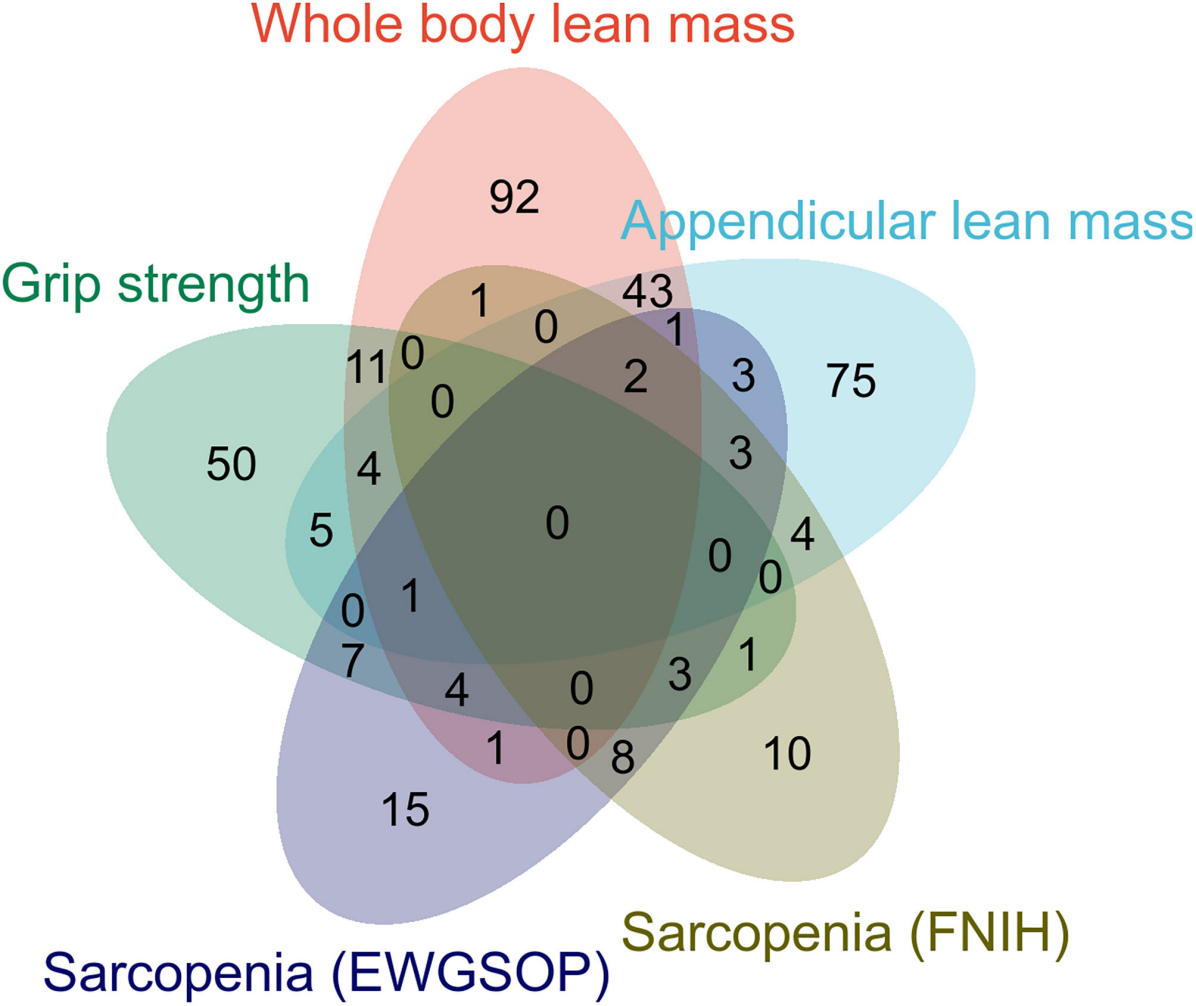
Wayne diagram of brain structural imaging indexes with significant causal effect on sarcopenia related traits. EWGSOP = European Working Group on Sarcopenia in Older People, FNIH = Foundations of the National Institutes of Health).

**FIGURE 3:**
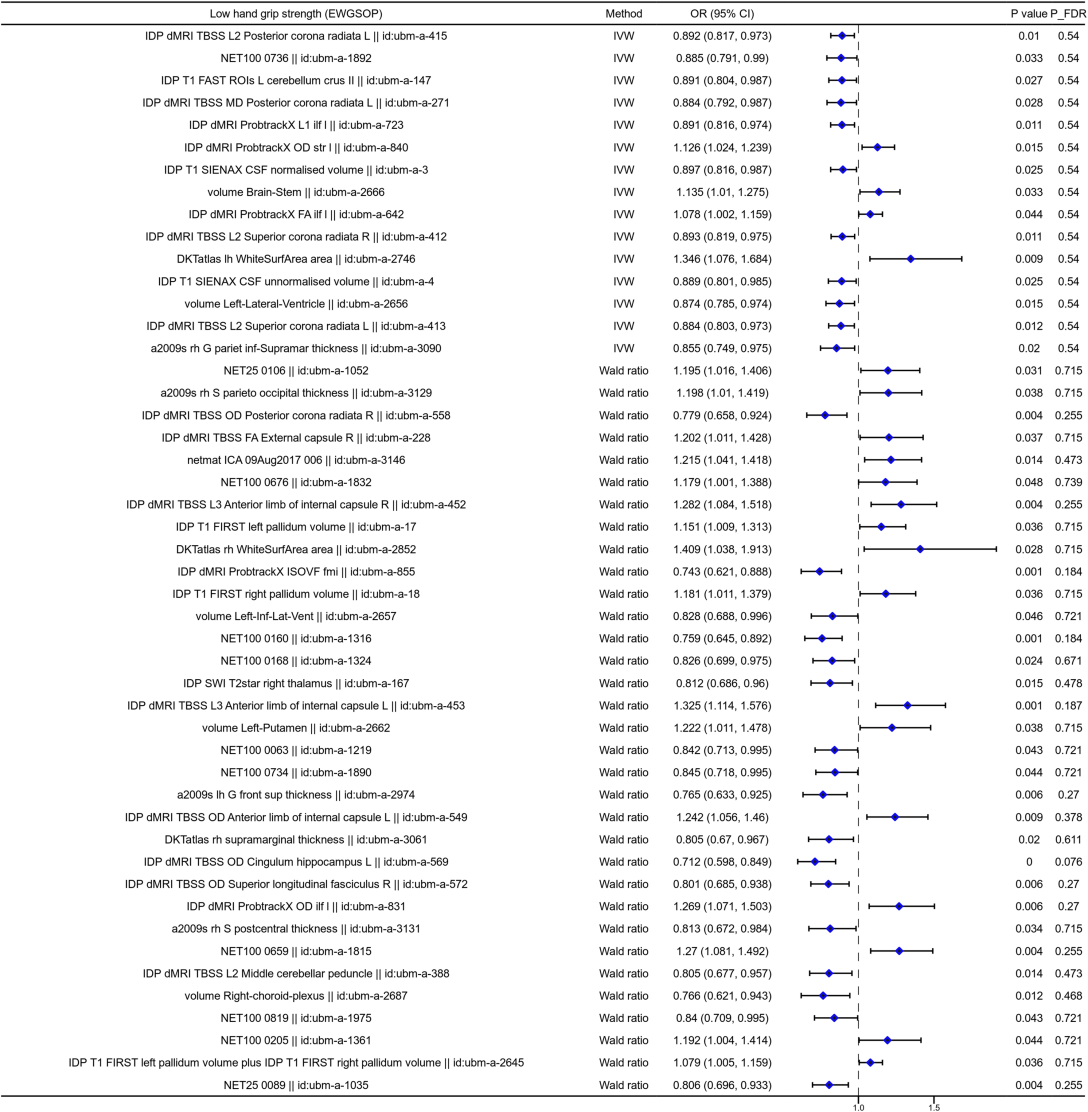
The forest diagram showing 48 brain structure imaging indexes with significantly causal effect on sarcopenia risk identified with EWGSOP criteria. EWGSOP = European Working Group on Sarcopenia in Older People.

**FIGURE 4:**
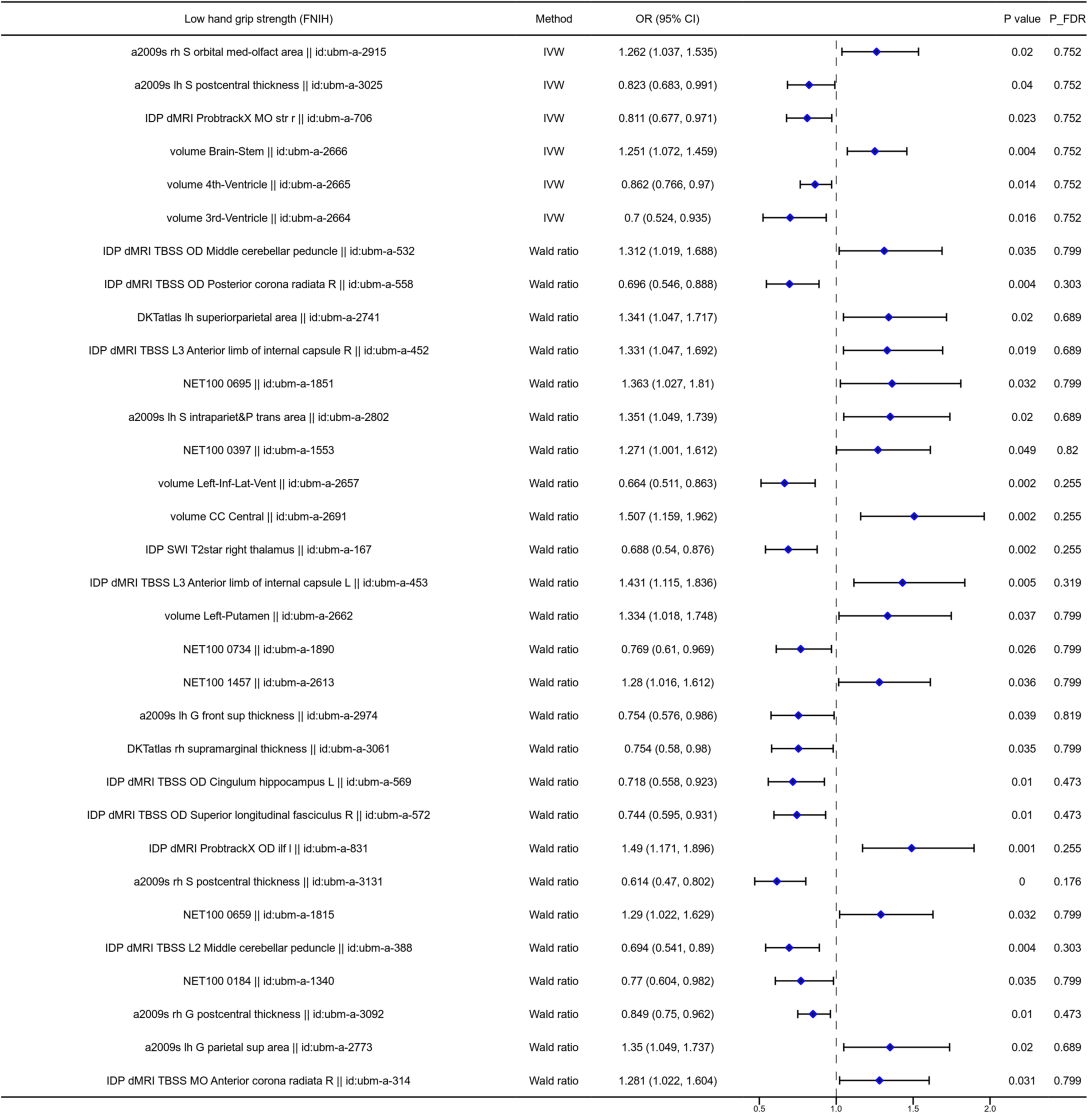
The forest diagram showing 32 brain structure imaging indexes with significantly causal effect on sarcopenia risk identified with FNIH criteria. FNIH = Foundations of the National Institutes of Health.

**FIGURE 5:**
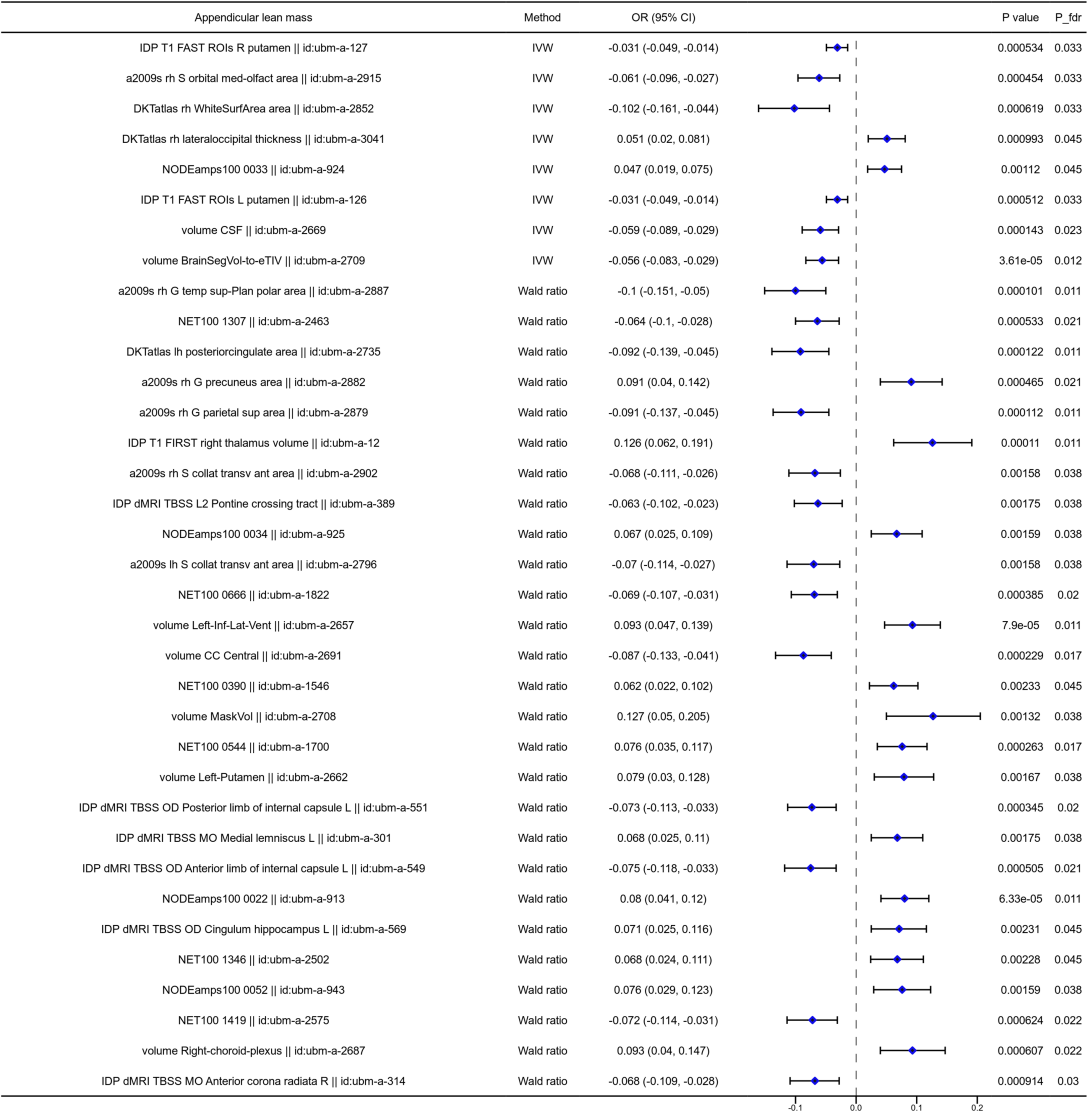
The forest diagram showing 35 brain structure imaging indexes with significantly causal effect on appendicular lean mass after FDR correction.

**FIGURE 6:**
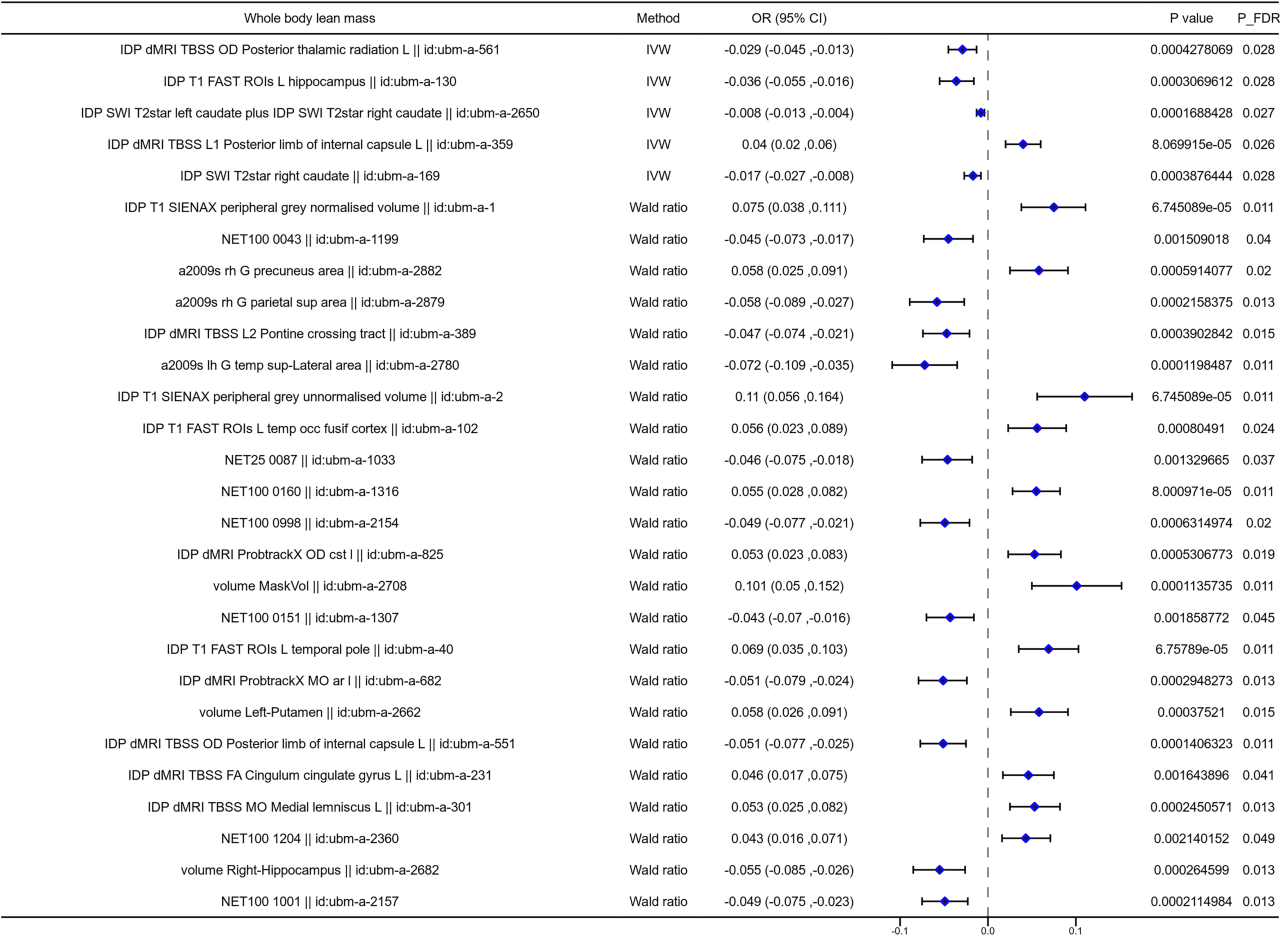
The forest diagram showing 28 brain structure imaging indexes with significantly causal effect on whole body lean mass after FDR correction.

**FIGURE 7:**
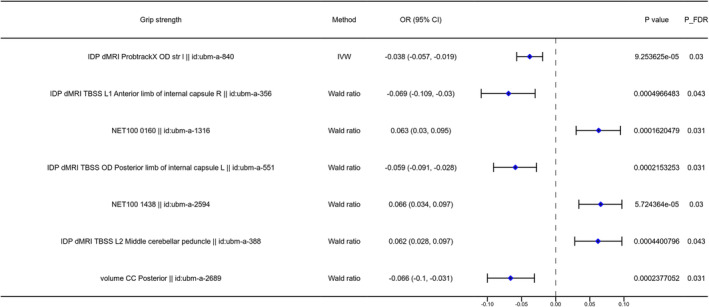
The forest diagram showing seven brain structure imaging indexes with significantly causal effect on left grip strength after FDR correction.

The two‐sample MR analysis was used to investigate the causal association between the brain structure changes of different regions and sarcopenia‐related traits, including ALM, WBLM, grip strength and sarcopenia diagnosed by the EWGSOP or FNIH criteria. For the causal association between BIDs and lean mass, as shown in Figure 2, there were 141 BIDs identified to show significant causal effect on ALM. For the causal association between BIDs and grip strength, we found 86 BIDs showing significantly causal effect on the grip strength of the left hand. And we found 160 BIDs showing significant causal effect on the WBLM. The MR analysis results of these three sarcopenia traits were listed in Tables S2–S4. We also investigated the causal association between BIDs and sarcopenia diagnosed by different criteria. The MR analysis results indicated that there were 48 BIDs identified to be causally associated with sarcopenia diagnosed by the EWGSOP criteria (Figure 3), and 32 BIDs identified to be causally associated with sarcopenia diagnosed by the FNIH criteria (Figure 4). And there were 44 BIDs identified to be causally associated with both the WBLM and ALM (Figure 2). And there were three BIDs identified to be associated with both the grip strength and sarcopenia (Figure 2). After FDR correction, we found that there were 35 BIDs still showing causal effect on the ALM (Figure 5), and there were 28 BIDs showing causal effect on the WBLM (Figure 6). In addition, there were 7 BIDs showing causal effect on grip strength (Figure 7), although no significant association between BIDs and sarcopenia was detected after FDR correction. The sensitivity analysis results of MR analysis between brain structure imaging indexes and ALM, grip strength, WBLM, sarcopenia diagnosed by the EWGSOP criteria and sarcopenia diagnosed by the FNIH criteria were listed in Tables S5–S9.

### 3.2 The Causal Effect of Gene Expression of Different Brain Regions on Sarcopenia

**FIGURE 8:**
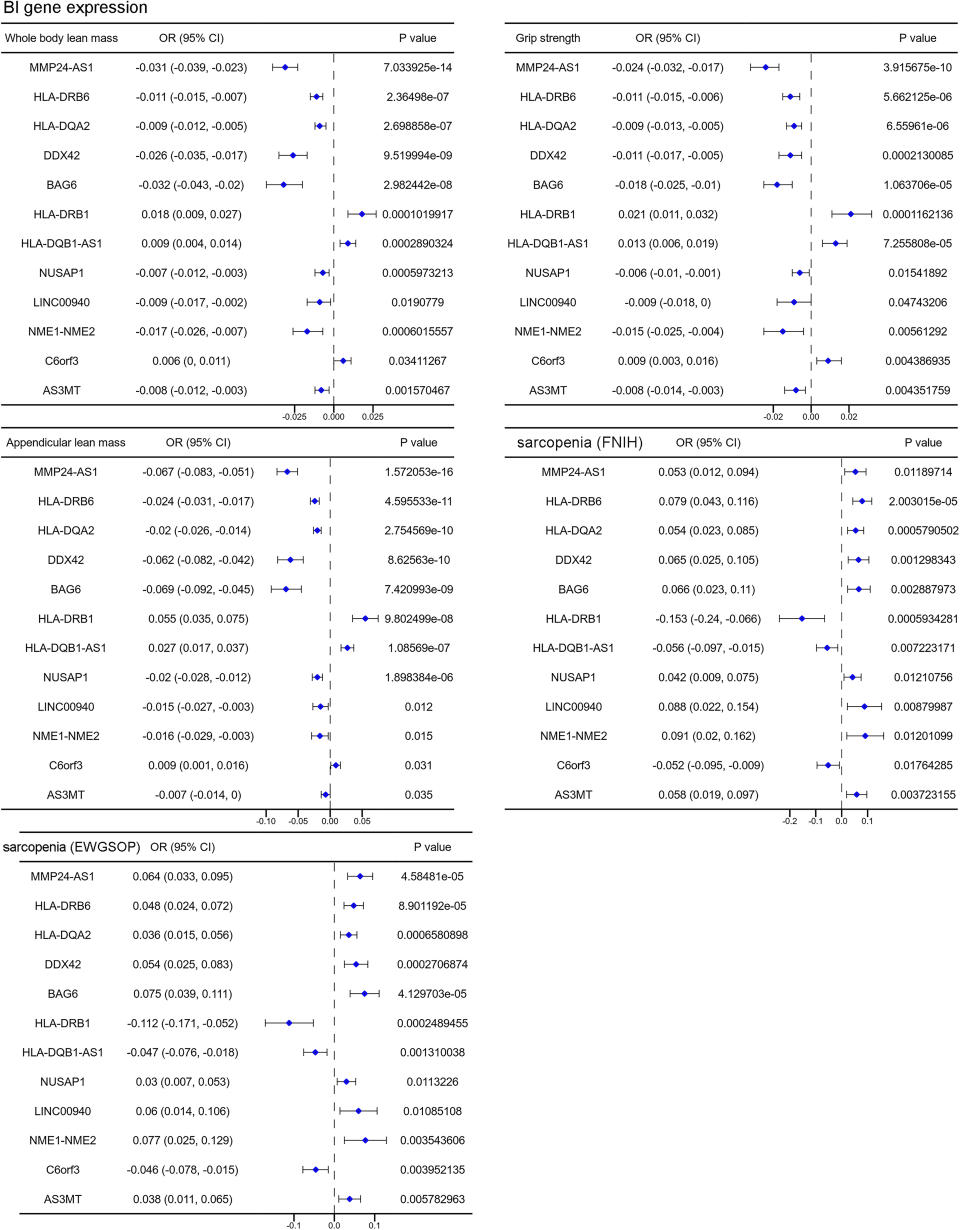
The forest diagram showing 12 gene in B1 region with significantly causal effect on all the 5 sarcopenia‐related traits.

We further investigated whether there were significantly causal association between the gene expression of different brain regions and sarcopenia‐related traits. We included 13 GWAS summary datasets of gene expression of 13 brain regions. As shown in Figure S1A, there were 12 genes expressed in the brain amygdala region showing causal effect on all the five sarcopenia‐related traits. The over‐expression of nine genes (MMP24‐AS1, HLA‐DRB6, HLA‐DQA2, DDX42, BAG6, NUSAP1, LINC00940, NME1‐NME2 and AS3MT) in the amygdala region showing detrimental effect on all the five sarcopenia‐related traits, whereas three genes (HLA‐DRB1, HLA‐DQB1‐AS1 and C6ORF3) showing protective effect (Figure 8). The function rich analysis (Figure S1B) indicated that the gene expression pattern (12 genes) in the brain amygdala region is enriched in immune‐related pathways, such as MHC protein complex assembly. In addition, we also predicted the potential transcription factors and interacting microRNA of these 12 genes (Figure S1C–E).

In the B2 region, as shown in Figure S2, we identified 16 genes that showing significantly causal association with all the five sarcopenia‐related traits, including 5 protective genes and 11 risk genes.

In the B3 region, as shown in Figure S3, we identified 36 genes that showing significantly causal association with all the five sarcopenia‐related traits, including 15 protective genes and 21 risk genes.

In the B4 region, as shown in Figure S4, we identified 40 genes that showing significantly causal association with all the five sarcopenia‐related traits, including 19 protective genes and 21 risk genes.

In the B5 region, as shown in Figure S5, we identified 48 genes that showing significantly causal association with all the five sarcopenia‐related traits, including 23 protective genes and 25 risk genes.

In the B6 region, as shown in Figure S6, we identified 37 genes that showing significantly causal association with all the five sarcopenia‐related traits, including 15 protective genes and 22 risk genes.

In the B7 region, as shown in Figure S7, we identified 29 genes that showing significantly causal association with all the five sarcopenia‐related traits, including 10 protective genes and 19 risk genes.

In the B8 region, as shown in Figure S8, we identified 20 genes that showing significantly causal association with all the five sarcopenia‐related traits, including 6 protective genes and 14 risk genes.

In the B9 region, as shown in Figure S9, we identified 23 genes that showing significantly causal association with all the five sarcopenia‐related traits, including 8 protective genes and 15 risk genes.

In the B10 region, as shown in Figure S10, we identified 31 genes that showing significantly causal association with all the five sarcopenia‐related traits, including 12 protective genes and 19 risk genes.

In the B11 region, as shown in Figure S11, we identified 25 genes that showing significantly causal association with all the five sarcopenia‐related traits, including nine protective genes and 16 risk genes.

In the B12 region, as shown in Figure S12, we identified 18 genes that showing significantly causal association with all the 5 sarcopenia‐related traits, including 3 protective genes and 15 risk genes.

In the B13 region, as shown in Figure S13, we identified 13 genes that showing significantly causal association with all the five sarcopenia‐related traits, including four protective genes and nine risk genes.

## 4 Discussion

The intertwined connections between muscle and brain are becoming increasing attention. In our study, the two‐sample MR analysis was used to investigate the causal association between the brain structure changes of different regions and sarcopenia‐related traits, including ALM, WBLM, grip strength and sarcopenia diagnosed by the EWGSOP or FNIH criteria. Besides, we included 13 GWAS summary datasets of gene expression of 13 brain regions, including amygdala, anterior cingulate cortex, the basal ganglia, cerebellum, the cerebellar hemisphere, the cerebral cortex, Brodmann area 9, hippocampus, hypothalamus, nucleus accumbens basal ganglia, the putamen basal ganglia, the spinal cord cervical and the substantia nigra and analysed the relationship between these genes and sarcopenia. According to our analysis, we revealed significant causal links between sarcopenia and brain structure and gene expression. Hence, we propose the possibility of the brain–muscle axis based on the significant relationship between brain and muscle‐related diseases.

To figure out the cause of sarcopenia, previous researches have revealed the association between muscle and brain functions [14, 15, 16, 17, 18]. However, it remained unclear how brain influenced muscle homeostasis. The analysis of cross‐sectional data revealed that ageing is an independent risk factor for sarcopenia [15] and ageing is a biological process that impacts numerous systems and organs [16], brain included as well. As we all know, the ageing process affects the brain in a number of ways, including changing its size, blood flow, neurological system and ability to think [17], which will then impact the activity of other organs and tissues. It is possible that ageing brains could influence the activity of muscle and induce occurrence of muscle‐related diseases. In our study, we assumed that brain‐muscle axis did exist and participate in the development of sarcopenia and wondered if brain structures and related genes expression could influence sarcopenia relevant traits.

We firstly analysed the relationship between brain structures and sarcopenia using brain imaging‐derived phenotypes, which have been regularly applied to analyse the association between brain structures and other diseases. For example, one psychiatric research used brain imaging–derived phenotypes and anxiety relevant data to find out that the right posterior middle cingulate gyrus, grey matter volume of the right anterior superior temporal gyrus and the right posterior middle cingulate gyrus were significantly associated with anxiety symptoms [18]. There are also studies using them to analyse the relationship between brain structures and cognitive impairment and Alzheimer's disease [19, 20]. In our study, we combined the brain imaging–derived phenotypes with MR analysis and discovered that many brain areas have significantly causal effects on sarcopenia traits, indicating that the brain had causal influence on development of sarcopenia.

Then we selected 13 brain areas to analyse the effect of brain‐related genes on sarcopenia, which showed causal relationships between these brain areas and sarcopenia. Among them, some areas are associated with motor control, such as the anterior cingulate cortex, basal ganglia, cerebellar hemisphere, Brodmann area 9 (BA9), nucleus accumbens (NAc) and putamen. Amygdala is linked to a variety of cognitive activities. The anterior cingulate cortex is activated in both acute and chronic pain, which is related with emotion, action and memory [21]. Many higher brain functions, such as memory, thinking, learning, reasoning, problem‐solving, emotions, consciousness and sensory processes, depend heavily on the cerebral cortex [22]. The temporal lobe contains the intricate brain structure known as the hippocampus, which is mostly involved in memory and learning [23]. The hypothalamus is responsible for maintaining internal body equilibrium and controlling different functions such as hormone production, body temperature, heart rate, appetite, mood and sleep [24]. The cervical spinal cord is a vital part of the central nervous system, responsible for transmitting sensory and motor signals between the brain and the rest of the body, particularly the upper extremities [25]. As part of the basal ganglia, the substantia nigra (SN) is a midbrain dopaminergic nucleus that modulates motor activity and reward processes [26]. However, most of these brain regions have not been reported to be associated with muscle function and are not well studied in the mechanism between their function and sarcopenia, which requires further experiments to explore and validate.

Next, we found that many identical genes were expressed in different regions. For example, MMP24‐AS1, HLA‐DQA2 and AS3MT were found in all brain structures. Research has shown that AS3MT is implicated in normal neurodevelopment and neurodevelopmental disorders such as schizophrenia [27, 28]. Additionally, studies using rodent models have demonstrated arsenic accumulation in the brain, leading to defects in operant learning, behavioural changes and affect [27]. Consistent with our study, the gene has been found to be ubiquitously expressed in the mouse brain, including the cerebral cortex, hippocampus and cerebellum, as well as in adult human neurons and astrocytes [29]. These findings suggest a potential role for AS3MT in brain function and development. MMP24‐AS1 is a noncoding RNA gene that is associated with readthrough transcription between upstream genes, and the readthrough transcripts may encode proteins similar to those encoded by the EDEM2 gene [30]. EDEM2 is involved in the endoplasmic reticulum‐associated degradation (ERAD) pathway, which is responsible for the degradation of misfolded proteins in the endoplasmic reticulum (ER) [31]. Furthermore, EDEM2 is also associated with immune infiltration in glioma and is considered a diagnostic and prognostic biomarker for the disease [32]. Studies have shown that EDEM2 silencing decreases SLC2A2 and PXD1 expression, leading to impaired insulin secretion, and that it is involved in early childhood diabetes [33]. In the context of the brain, EDEM2 has been found to be expressed in the thalamus [32]. Additionally, high expression of synoviolin (SYVN1)/HRD1 and EDEM2, which targets glycoproteins for ERAD, have been associated with brain‐related conditions [34]. Research has shown that HLA‐DQA2 is expressed on the surface of B lymphoblastoid cells and is specifically expressed in human Langerhans cells [35]. Additionally, studies have suggested an association between HLA‐DQA2 and Moyamoya disease in the Chinese Han population [36]. Consistent with our study, HLA‐DQA2 has been implicated in susceptibility to myasthenia gravis [37]. Although the role of these genes in sarcopenia and muscle function has not been studied, some of them such as AS3MT and EDEM2 have been reported to affect brain function and development. Further studies are still required to investigate their roles in muscle development and verify the mechanisms between their function and brain‐muscle axis.

There are various advantages to our study. This is the first study to apply MR analysis to investigate the genetic and causal relationships between brain structures and sarcopenia. We showed the potential link between brain structures and muscle‐related diseases and originally proposed the concept of the brain–muscle axis, which provided theoretical support for future brain–muscle axis relevant research. Furthermore, our findings could also provide a new understanding of the development of sarcopenia and indicate the importance of brain structure and function in muscle ability, which will help clinicians to manage sarcopenia patients from a new aspect, and also provide new potential therapies for sarcopenia patients. Besides, MR analysis can minimize the potential for confounding factors and reverse causation, according to its principle. Due to the large sample sizes of GWAS, the winner's curse or weak instruments may be reduced, which may result in better levels of statistical power. However, it is inevitable that there were still some disadvantages in this study. Firstly, due to our study's population only including Europeans, the conclusion could be restricted to applying to the other population. Secondly, although we evaluated multiplicity using many techniques, it was not possible to exclude it entirely. Thirdly, there was a rather low incidence of sarcopenia. Larger populations need to be used for additional confirmation. Most importantly, because most of the significant brain structures and genes are firstly found to be genetically associated with muscle function and sarcopenia in our study, a lot of specific experiments and clinical studies are still required to verify their function and clarify their importance in brain‐muscle axis, which requires more time, foundations and better‐designed studies to complete.

In conclusion, our results are consistent with a possible causal link between sarcopenia and brain structure. We discovered the causal effect of different BIDs and brain region gene expression on ALM, WBLM, grip strength and sarcopenia diagnosed by EWGSOP or FNIH. The findings point to a significant relationship between brain and muscle‐related diseases, which is proposed as the brain–muscle axis in our study. Besides, the positive genes were mainly enriched in immune‐related signalling, suggesting that there may be an immune‐based cross‐organ regulation mechanism of the brain–muscle axis. However, our study only reported the potential genetic connections and causal relationships at the genetic level. More research into the underlying systems is needed to validate this biological explanation.

## Conflicts of Interest

The authors declare no conflicts of interest.

## Supporting information


**Figure S1** The causal effect of gene expression in brain amygdala region (B1) on sarcopenia‐related traits. (A) Wayne diagram of brain amygdala gene expression with significant causal effect on sarcopenia related traits; (B) the gene signaling enriched by the genes expressed in brain amygdala region and showing significantly causal effect on all the 5 sarcopenia‐related traits; (C) the protein‐protein interaction (PPI) network of screened genes showing significantly causal effect on all the 5 sarcopenia‐related traits; (D) the potential translational factors interacted with screened genes showing significantly causal effect on all the 5 sarcopenia‐related traits; (E) the potential miRNA interacted with screened genes showing significantly causal effect on all the 5 sarcopenia‐related traits。
**Figure S2.** The causal effect of gene expression in Brain Anterior cingulate cortex BA24 region (B2) on sarcopenia‐related traits: (A) Wayne diagram of B2 region gene expression with significant causal effect on sarcopenia related traits; (B) the gene signaling enriched by the genes expressed in B2 region and showing significantly causal effect on all the 5 sarcopenia‐related traits; (C) the protein‐protein interaction (PPI) network of screened genes showing significantly causal effect on all the 5 sarcopenia‐related traits; (D) the potential translational factors interacted with screened genes showing significantly causal effect on all the 5 sarcopenia‐related traits; (E) the potential miRNA interacted with screened genes showing significantly causal effect on all the 5 sarcopenia‐related traits; (F) the forest diagram showing 12 gene in B2 region with significantly causal effect on all the 5 sarcopenia‐related traits.
**Figure S3.** The causal effect of gene expression in Brain Caudate basal ganglia region (B3) on sarcopenia‐related traits: (A) Wayne diagram of B3 region gene expression with significant causal effect on sarcopenia related traits; (B) the gene signaling enriched by the genes expressed in B3 region and showing significantly causal effect on all the 5 sarcopenia‐related traits; (C) the protein‐protein interaction (PPI) network of screened genes showing significantly causal effect on all the 5 sarcopenia‐related traits; (D) the potential translational factors interacted with screened genes showing significantly causal effect on all the 5 sarcopenia‐related traits; (E) the potential miRNA interacted with screened genes showing significantly causal effect on all the 5 sarcopenia‐related traits; (F) the forest diagram showing 12 gene in B3 region with significantly causal effect on all the 5 sarcopenia‐related traits.
**Figure S4.** The causal effect of gene expression in Brain Cerebellar Hemisphere region (B4) on sarcopenia‐related traits: (A) Wayne diagram of B4 region gene expression with significant causal effect on sarcopenia related traits; (B) the gene signaling enriched by the genes expressed in B4 region and showing significantly causal effect on all the 5 sarcopenia‐related traits; (C) the protein‐protein interaction (PPI) network of screened genes showing significantly causal effect on all the 5 sarcopenia‐related traits; (D) the potential translational factors interacted with screened genes showing significantly causal effect on all the 5 sarcopenia‐related traits; (E) the potential miRNA interacted with screened genes showing significantly causal effect on all the 5 sarcopenia‐related traits; (F) the forest diagram showing 12 gene in B4 region with significantly causal effect on all the 5 sarcopenia‐related traits.
**Figure S5.** The causal effect of gene expression in Brain Cerebellum region (B5) on sarcopenia‐related traits: (A) Wayne diagram of B5 region gene expression with significant causal effect on sarcopenia related traits; (B) the gene signaling enriched by the genes expressed in B5 region and showing significantly causal effect on all the 5 sarcopenia‐related traits; (C) the protein‐protein interaction (PPI) network of screened genes showing significantly causal effect on all the 5 sarcopenia‐related traits; (D) the potential translational factors interacted with screened genes showing significantly causal effect on all the 5 sarcopenia‐related traits; (E) the potential miRNA interacted with screened genes showing significantly causal effect on all the 5 sarcopenia‐related traits; (F) the forest diagram showing 12 gene in B5 region with significantly causal effect on all the 5 sarcopenia‐related traits.
**Figure S6.** The causal effect of gene expression in Brain Cortex (B6) on sarcopenia‐related traits: (A) Wayne diagram of B6 region gene expression with significant causal effect on sarcopenia related traits; (B) the gene signaling enriched by the genes expressed in B6 region and showing significantly causal effect on all the 5 sarcopenia‐related traits; (C) the protein‐protein interaction (PPI) network of screened genes showing significantly causal effect on all the 5 sarcopenia‐related traits; (D) the potential translational factors interacted with screened genes showing significantly causal effect on all the 5 sarcopenia‐related traits; (E) the potential miRNA interacted with screened genes showing significantly causal effect on all the 5 sarcopenia‐related traits; (F) the forest diagram showing 12 gene in B6 region with significantly causal effect on all the 5 sarcopenia‐related traits.
**Figure S7.** The causal effect of gene expression in Brain Frontal Cortex BA9 region (B7) on sarcopenia‐related traits: (A) Wayne diagram of B7 region gene expression with significant causal effect on sarcopenia related traits; (B) the gene signaling enriched by the genes expressed in B7 region and showing significantly causal effect on all the 5 sarcopenia‐related traits; (C) the protein‐protein interaction (PPI) network of screened genes showing significantly causal effect on all the 5 sarcopenia‐related traits; (D) the potential translational factors interacted with screened genes showing significantly causal effect on all the 5 sarcopenia‐related traits; (E) the potential miRNA interacted with screened genes showing significantly causal effect on all the 5 sarcopenia‐related traits; (F) the forest diagram showing 12 gene in B7 region with significantly causal effect on all the 5 sarcopenia‐related traits.
**Figure S8.** The causal effect of gene expression in Brain Hippocampus region (B8) on sarcopenia‐related traits: (A) Wayne diagram of B8 region gene expression with significant causal effect on sarcopenia related traits; (B) the gene signaling enriched by the genes expressed in B8 region and showing significantly causal effect on all the 5 sarcopenia‐related traits; (C) the protein‐protein interaction (PPI) network of screened genes showing significantly causal effect on all the 5 sarcopenia‐related traits; (D) the potential translational factors interacted with screened genes showing significantly causal effect on all the 5 sarcopenia‐related traits; (E) the potential miRNA interacted with screened genes showing significantly causal effect on all the 5 sarcopenia‐related traits; (F) the forest diagram showing 12 gene in B8 region with significantly causal effect on all the 5 sarcopenia‐related traits.
**Figure S9.** The causal effect of gene expression in Brain Hypothalamus region (B9) on sarcopenia‐related traits: (A) Wayne diagram of B9 region gene expression with significant causal effect on sarcopenia related traits; (B) the gene signaling enriched by the genes expressed in B9 region and showing significantly causal effect on all the 5 sarcopenia‐related traits; (C) the protein‐protein interaction (PPI) network of screened genes showing significantly causal effect on all the 5 sarcopenia‐related traits; (D) the potential translational factors interacted with screened genes showing significantly causal effect on all the 5 sarcopenia‐related traits; (E) the potential miRNA interacted with screened genes showing significantly causal effect on all the 5 sarcopenia‐related traits; (F) the forest diagram showing 12 gene in B9 region with significantly causal effect on all the 5 sarcopenia‐related traits.
**Figure S10.** The causal effect of gene expression in Brain Nucleus accumbens basal ganglia (B10) on sarcopenia‐related traits: (A) Wayne diagram of B10 region gene expression with significant causal effect on sarcopenia related traits; (B) the gene signaling enriched by the genes expressed in B10 region and showing significantly causal effect on all the 5 sarcopenia‐related traits; (C) the protein‐protein interaction (PPI) network of screened genes showing significantly causal effect on all the 5 sarcopenia‐related traits; (D) the potential translational factors interacted with screened genes showing significantly causal effect on all the 5 sarcopenia‐related traits; (E) the potential miRNA interacted with screened genes showing significantly causal effect on all the 5 sarcopenia‐related traits; (F) the forest diagram showing 12 gene in B10 region with significantly causal effect on all the 5 sarcopenia‐related traits.
**Figure S11.** The causal effect of gene expression in Brain Putamen basal ganglia region (B11) on sarcopenia‐related traits: (A) Wayne diagram of B11 region gene expression with significant causal effect on sarcopenia related traits; (B) the gene signaling enriched by the genes expressed in B11 region and showing significantly causal effect on all the 5 sarcopenia‐related traits; (C) the protein‐protein interaction (PPI) network of screened genes showing significantly causal effect on all the 5 sarcopenia‐related traits; (D) the potential translational factors interacted with screened genes showing significantly causal effect on all the 5 sarcopenia‐related traits; (E) the potential miRNA interacted with screened genes showing significantly causal effect on all the 5 sarcopenia‐related traits; (F) the forest diagram showing 12 gene in B11 region with significantly causal effect on all the 5 sarcopenia‐related traits.
**Figure S12.** The causal effect of gene expression in Brain Spinal cord cervical c‐1 region (B12) on sarcopenia‐related traits: (A) Wayne diagram of B12 region gene expression with significant causal effect on sarcopenia related traits; (B) the gene signaling enriched by the genes expressed in B12 region and showing significantly causal effect on all the 5 sarcopenia‐related traits; (C) the protein‐protein interaction (PPI) network of screened genes showing significantly causal effect on all the 5 sarcopenia‐related traits; (D) the potential translational factors interacted with screened genes showing significantly causal effect on all the 5 sarcopenia‐related traits; (E) the potential miRNA interacted with screened genes showing significantly causal effect on all the 5 sarcopenia‐related traits; (F) the forest diagram showing 12 gene in B12 region with significantly causal effect on all the 5 sarcopenia‐related traits.
**Figure S13.** The causal effect of gene expression in Brain Substantia nigra region (B13) on sarcopenia‐related traits: (A) Wayne diagram of B13 region gene expression with significant causal effect on sarcopenia related traits; (B) the gene signaling enriched by the genes expressed in B13 region and showing significantly causal effect on all the 5 sarcopenia‐related traits; (C) the protein‐protein interaction (PPI) network of screened genes showing significantly causal effect on all the 5 sarcopenia‐related traits; (D) the potential translational factors interacted with screened genes showing significantly causal effect on all the 5 sarcopenia‐related traits; (E) the potential miRNA interacted with screened genes showing significantly causal effect on all the 5 sarcopenia‐related traits; (F) the forest diagram showing 12 gene in B13 region with significantly causal effect on all the 5 sarcopenia‐related traits.

**Table S1** The baseline information of GWAS summary data related to sarcopenia‐related outcomes.
**Table S2.** The mendelian randomization analysis results showing 141 brain structure imaging indexes with significantly casual effect on appendicular lean mass.
**Table S3.** The mendelian randomization analysis results showing 86 brain structure imaging indexes with significantly casual effect on grip strength.
**Table S4.** The mendelian randomization analysis results showing 160 brain structure imaging indexes with significantly casual effect on whole body lean mass.
**Table S5.** The sensitivity analysis results of mendelian randomization analysis between brain structure imaging indexes and appendicular lean mass.
**Table S6.** The sensitivity analysis results of mendelian randomization analysis between brain structure imaging indexes and grip strength.
**Table S7.** The sensitivity analysis results of mendelian randomization analysis between brain structure imaging indexes and whole body lean mass.
**Table S8.** The sensitivity analysis results of mendelian randomization analysis between brain structure imaging indexes and sarcopenia identified by EWGSOP criteria.
**Table S9.** The sensitivity analysis results of mendelian randomization analysis between brain structure imaging indexes and identified by FNIH criteria.
