# Supplementary material for: Genetic Influence of the Brain on Muscle Structure: A Mendelian Randomization Study of Sarcopenia
Source: J Cachexia Sarcopenia Muscle. 2024 Nov 13;16(1):e13647. doi: 10.1002/jcsm.13647 (PMC11695463; doi:10.1002/jcsm.13647)
Supplement: Supplementary file 1 — Figure S1 The causal effect of gene expression in brain amygdala region (B1) on sarcopenia‐related traits. (A) Wayne diagram of brain amygdala gene expression with significant causal effect on sarcopenia related traits; (B) the gene signaling enriched by the genes expressed in brain amygdala region and showing significantly causal effect on all the 5 sarcopenia‐related traits; (C) the protein‐protein interaction (PPI) network of screened genes showing significantly causal effect on all the 5 sarcopenia‐related traits; (D) the potential translational factors interacted with screened genes showing significantly causal effect on all the 5 sarcopenia‐related traits; (E) the potential miRNA interacted with screened genes showing significantly causal effect on all the 5 sarcopenia‐related traits。 Figure S2. The causal effect of gene expression in Brain Anterior cingulate cortex BA24 region (B2) on sarcopenia‐related traits: (A) Wayne diagram of B2 region gene expression with significant causal effect on sarcopenia related traits; (B) the gene signaling enriched by the genes expressed in B2 region and showing significantly causal effect on all the 5 sarcopenia‐related traits; (C) the protein‐protein interaction (PPI) network of screened genes showing significantly causal effect on all the 5 sarcopenia‐related traits; (D) the potential translational factors interacted with screened genes showing significantly causal effect on all the 5 sarcopenia‐related traits; (E) the potential miRNA interacted with screened genes showing significantly causal effect on all the 5 sarcopenia‐related traits; (F) the forest diagram showing 12 gene in B2 region with significantly causal effect on all the 5 sarcopenia‐related traits. Figure S3. The causal effect of gene expression in Brain Caudate basal ganglia region (B3) on sarcopenia‐related traits: (A) Wayne diagram of B3 region gene expression with significant causal effect on sarcopenia related traits; (B) the gene signaling enriched by t [file JCSM-16-e13647-s002.docx]

**Supplementary Figures**

**
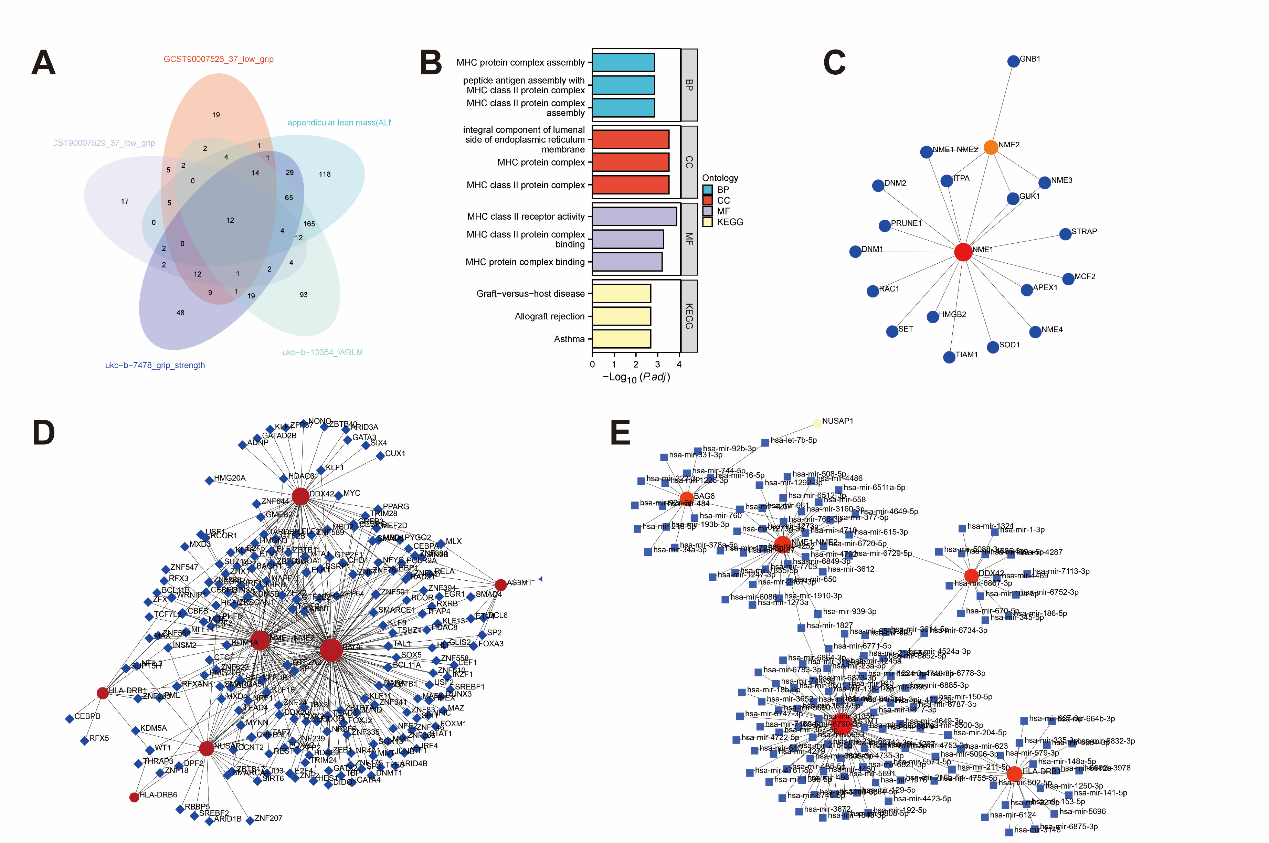
**

**Figure S1.** The causal effect of gene expression in brain amygdala region (B1) on sarcopenia-related traits. (A) Wayne diagram of brain amygdala gene expression with significant causal effect on sarcopenia related traits; (B) the gene signaling enriched by the genes expressed in brain amygdala region and showing significantly causal effect on all the 5 sarcopenia-related traits; (C) the protein-protein interaction (PPI) network of screened genes showing significantly causal effect on all the 5 sarcopenia-related traits; (D) the potential translational factors interacted with screened genes showing significantly causal effect on all the 5 sarcopenia-related traits; (E) the potential miRNA interacted with screened genes showing significantly causal effect on all the 5 sarcopenia-related traits。


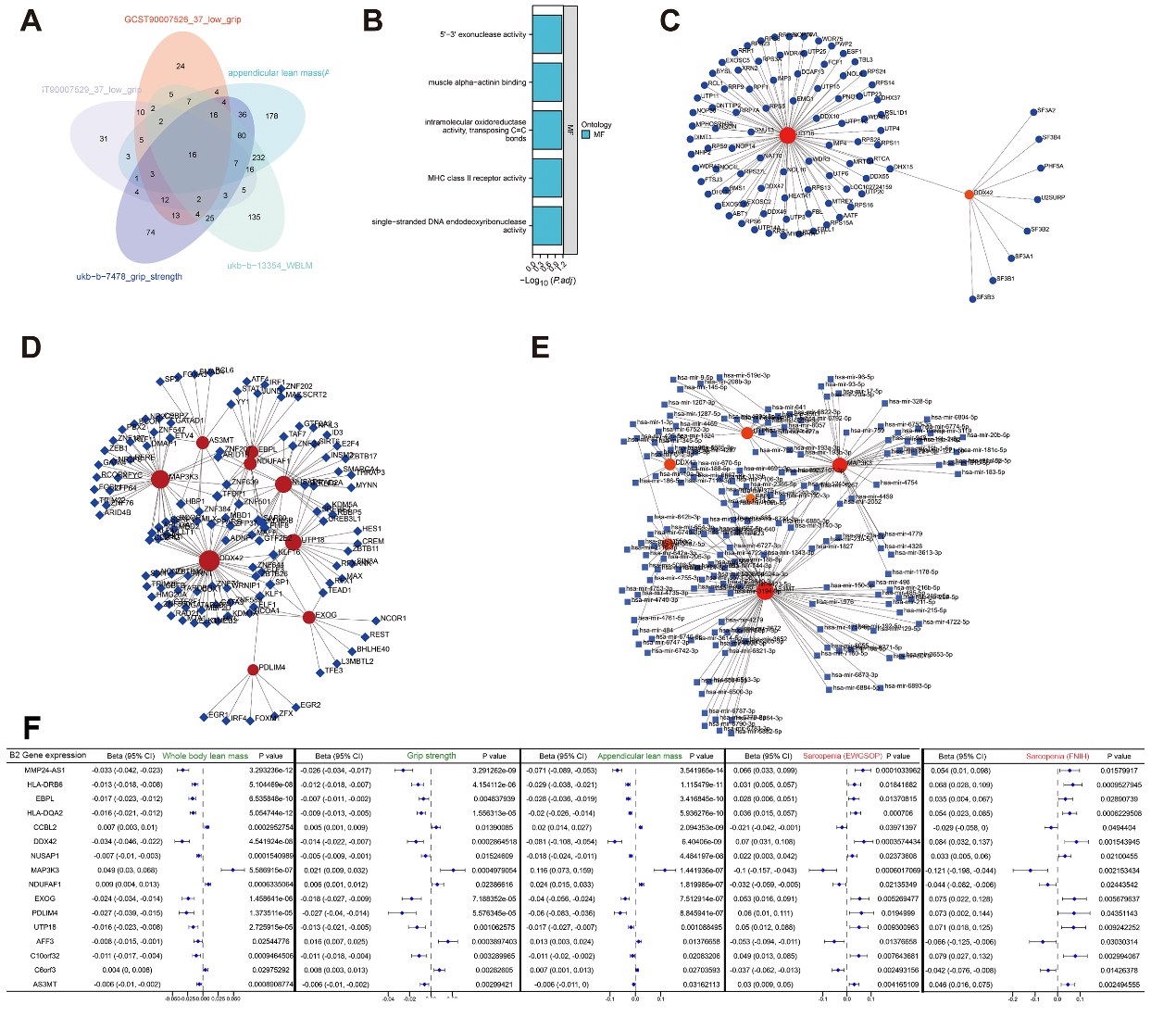


**Figure S2.** The causal effect of gene expression in Brain Anterior cingulate cortex BA24 region (B2) on sarcopenia-related traits: (A) Wayne diagram of B2 region gene expression with significant causal effect on sarcopenia related traits; (B) the gene signaling enriched by the genes expressed in B2 region and showing significantly causal effect on all the 5 sarcopenia-related traits; (C) the protein-protein interaction (PPI) network of screened genes showing significantly causal effect on all the 5 sarcopenia-related traits; (D) the potential translational factors interacted with screened genes showing significantly causal effect on all the 5 sarcopenia-related traits; (E) the potential miRNA interacted with screened genes showing significantly causal effect on all the 5 sarcopenia-related traits; (F) the forest diagram showing 12 gene in B2 region with significantly causal effect on all the 5 sarcopenia-related traits.


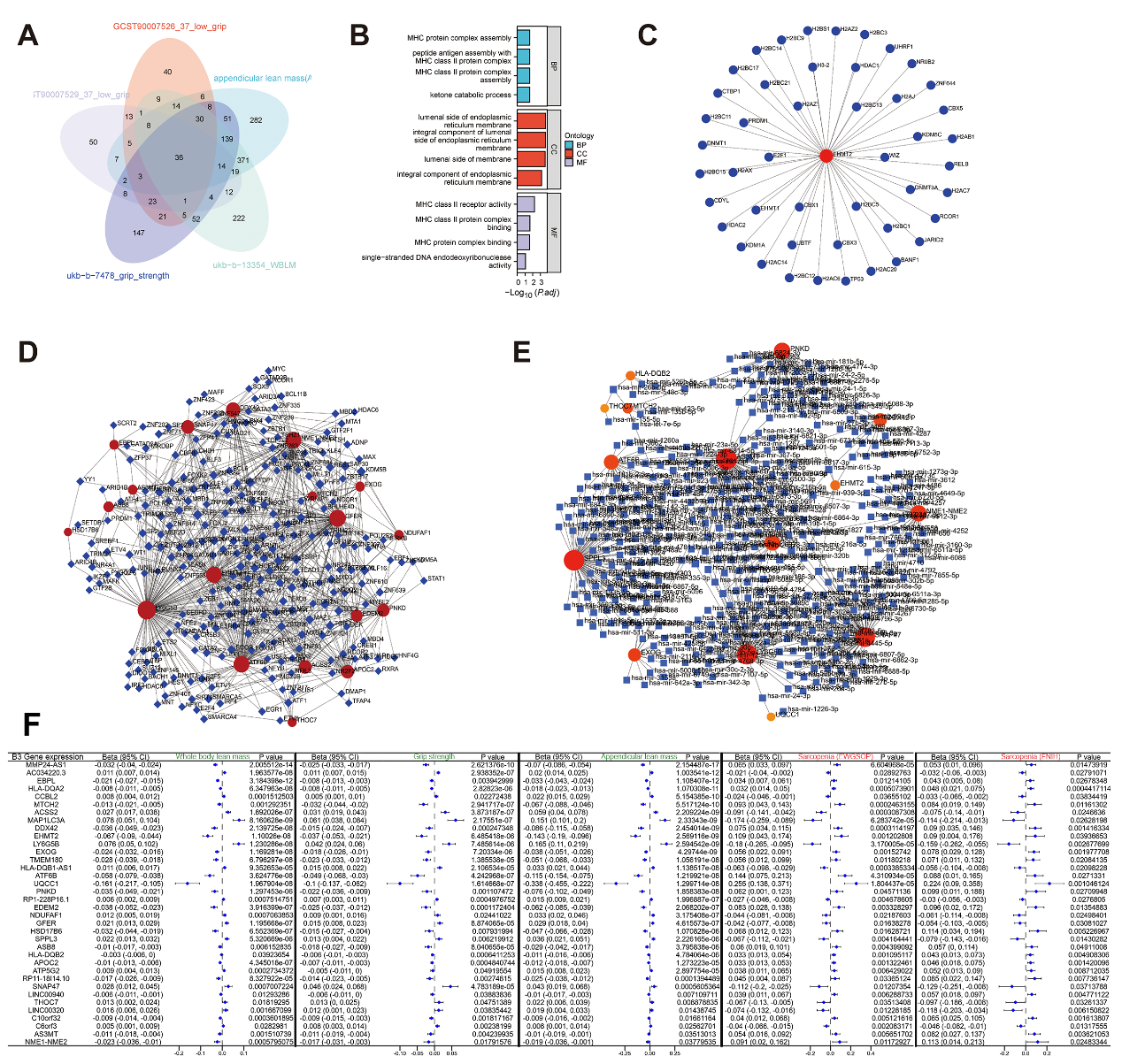


**Figure S3.** The causal effect of gene expression in Brain Caudate basal ganglia region (B3) on sarcopenia-related traits: (A) Wayne diagram of B3 region gene expression with significant causal effect on sarcopenia related traits; (B) the gene signaling enriched by the genes expressed in B3 region and showing significantly causal effect on all the 5 sarcopenia-related traits; (C) the protein-protein interaction (PPI) network of screened genes showing significantly causal effect on all the 5 sarcopenia-related traits; (D) the potential translational factors interacted with screened genes showing significantly causal effect on all the 5 sarcopenia-related traits; (E) the potential miRNA interacted with screened genes showing significantly causal effect on all the 5 sarcopenia-related traits; (F) the forest diagram showing 12 gene in B3 region with significantly causal effect on all the 5 sarcopenia-related traits.


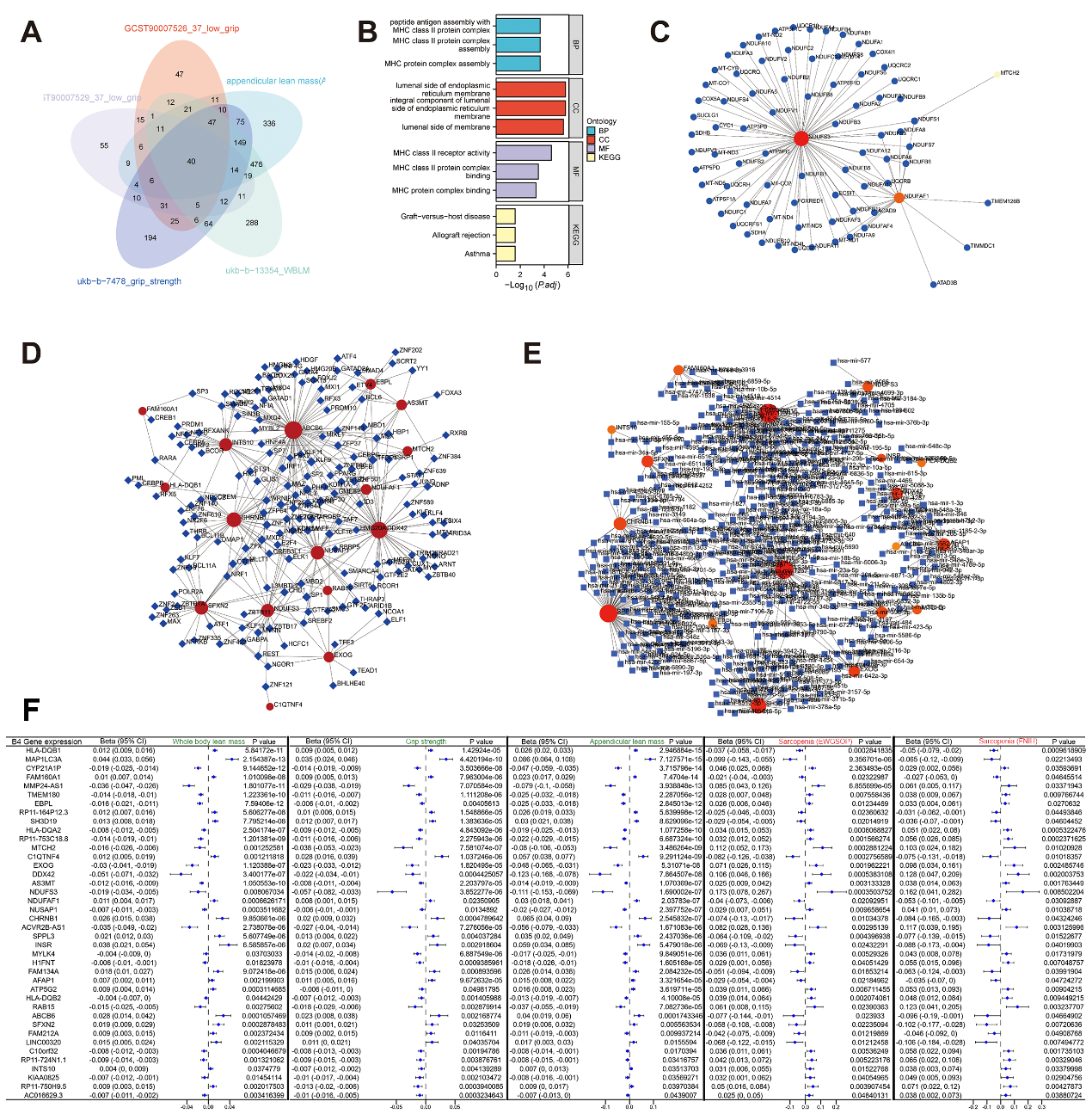


**Figure S4.** The causal effect of gene expression in Brain Cerebellar Hemisphere region (B4) on sarcopenia-related traits: (A) Wayne diagram of B4 region gene expression with significant causal effect on sarcopenia related traits; (B) the gene signaling enriched by the genes expressed in B4 region and showing significantly causal effect on all the 5 sarcopenia-related traits; (C) the protein-protein interaction (PPI) network of screened genes showing significantly causal effect on all the 5 sarcopenia-related traits; (D) the potential translational factors interacted with screened genes showing significantly causal effect on all the 5 sarcopenia-related traits; (E) the potential miRNA interacted with screened genes showing significantly causal effect on all the 5 sarcopenia-related traits; (F) the forest diagram showing 12 gene in B4 region with significantly causal effect on all the 5 sarcopenia-related traits.


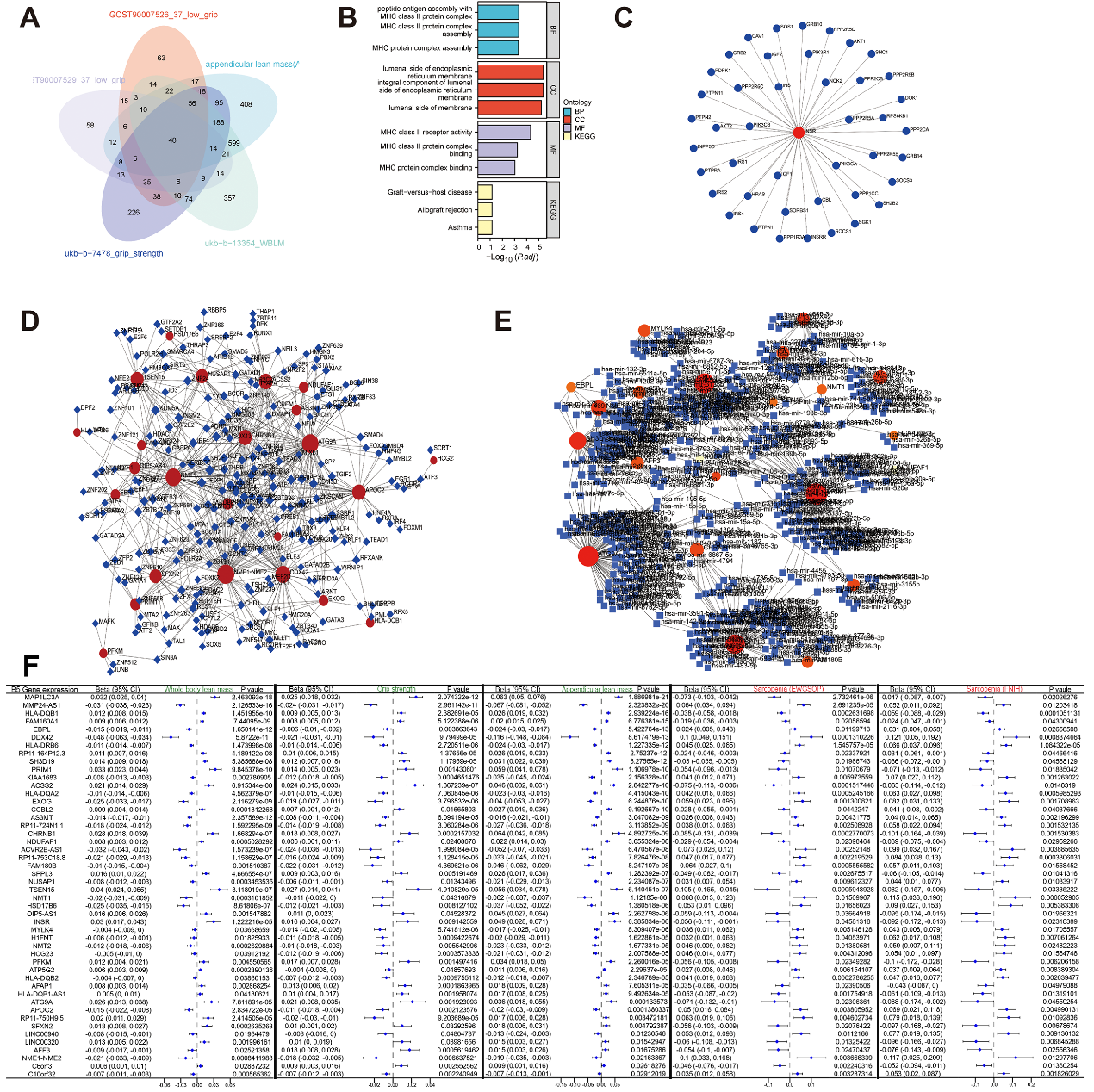


**Figure S5.** The causal effect of gene expression in [Brain Cerebellum](https://yanglab.westlake.edu.cn/data/SMR/GTEx_V8_cis_eqtl_summary/Brain_Cerebellum.zip) region (B5) on sarcopenia-related traits: (A) Wayne diagram of B5 region gene expression with significant causal effect on sarcopenia related traits; (B) the gene signaling enriched by the genes expressed in B5 region and showing significantly causal effect on all the 5 sarcopenia-related traits; (C) the protein-protein interaction (PPI) network of screened genes showing significantly causal effect on all the 5 sarcopenia-related traits; (D) the potential translational factors interacted with screened genes showing significantly causal effect on all the 5 sarcopenia-related traits; (E) the potential miRNA interacted with screened genes showing significantly causal effect on all the 5 sarcopenia-related traits; (F) the forest diagram showing 12 gene in B5 region with significantly causal effect on all the 5 sarcopenia-related traits.


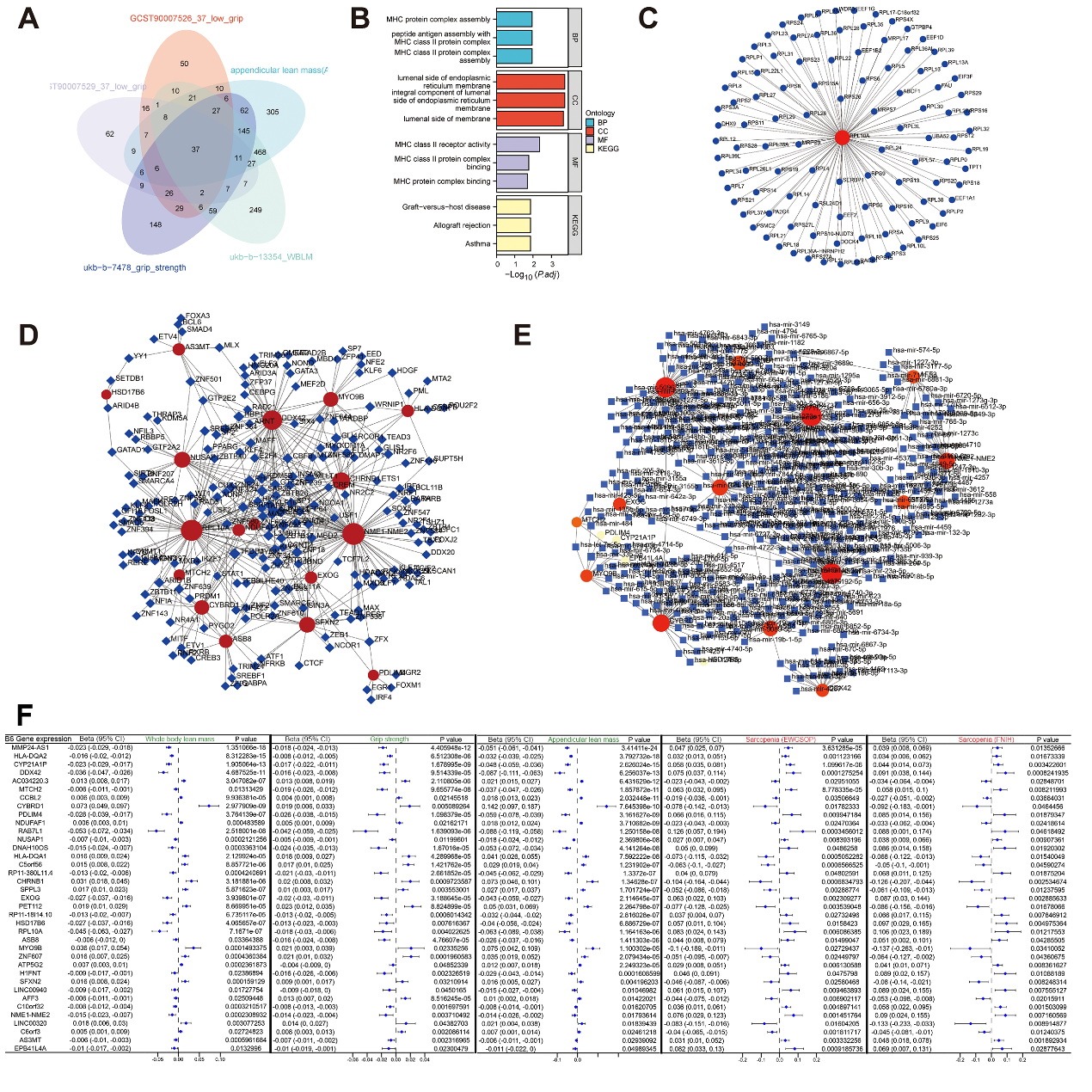


**Figure S6.** The causal effect of gene expression in [Brain Cortex](https://yanglab.westlake.edu.cn/data/SMR/GTEx_V8_cis_eqtl_summary/Brain_Cortex.zip) (B6) on sarcopenia-related traits: (A) Wayne diagram of B6 region gene expression with significant causal effect on sarcopenia related traits; (B) the gene signaling enriched by the genes expressed in B6 region and showing significantly causal effect on all the 5 sarcopenia-related traits; (C) the protein-protein interaction (PPI) network of screened genes showing significantly causal effect on all the 5 sarcopenia-related traits; (D) the potential translational factors interacted with screened genes showing significantly causal effect on all the 5 sarcopenia-related traits; (E) the potential miRNA interacted with screened genes showing significantly causal effect on all the 5 sarcopenia-related traits; (F) the forest diagram showing 12 gene in B6 region with significantly causal effect on all the 5 sarcopenia-related traits.


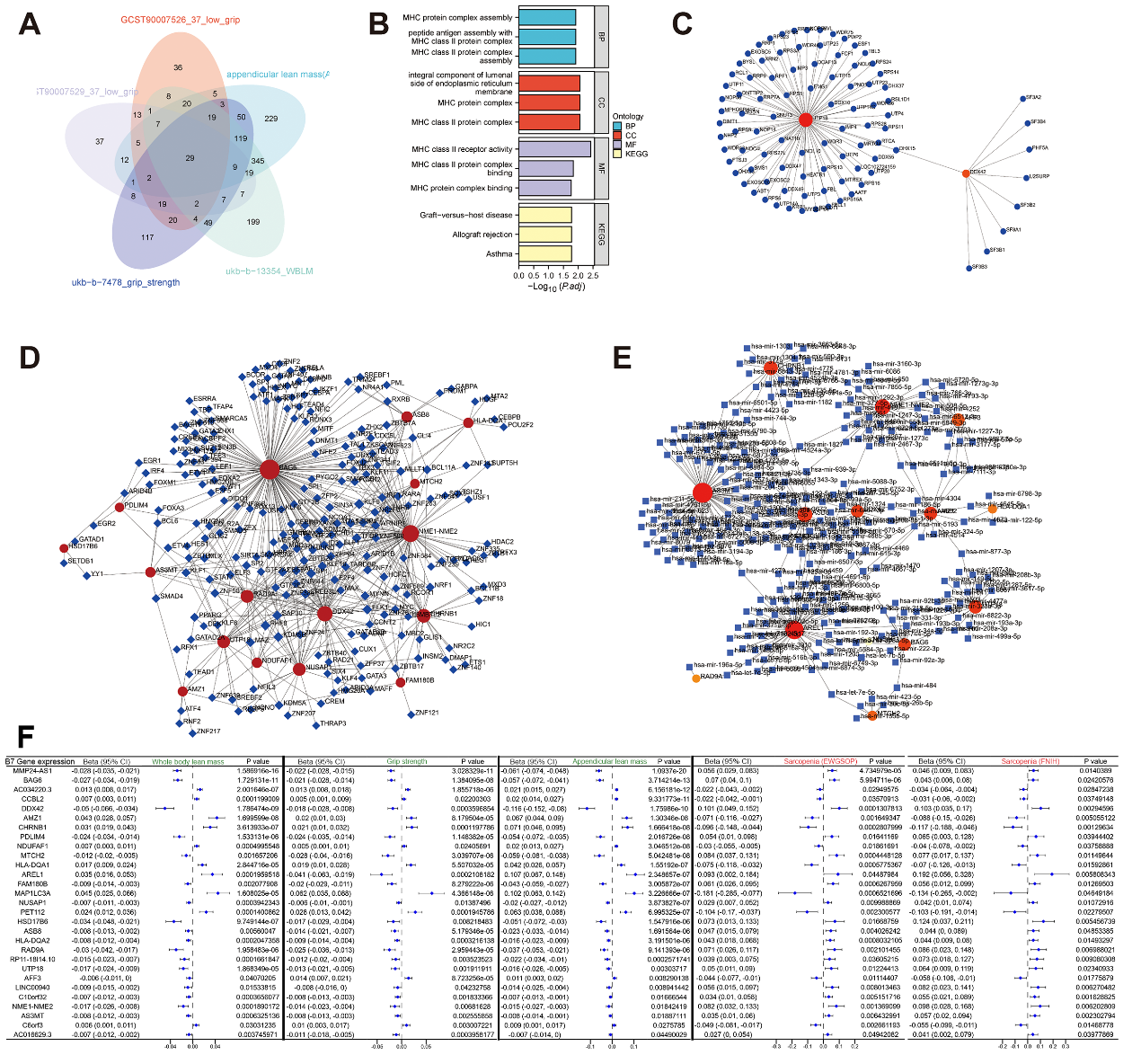


**Figure S7.** The causal effect of gene expression in [Brain Frontal Cortex BA9](https://yanglab.westlake.edu.cn/data/SMR/GTEx_V8_cis_eqtl_summary/Brain_Frontal_Cortex_BA9.zip) region (B7) on sarcopenia-related traits: (A) Wayne diagram of B7 region gene expression with significant causal effect on sarcopenia related traits; (B) the gene signaling enriched by the genes expressed in B7 region and showing significantly causal effect on all the 5 sarcopenia-related traits; (C) the protein-protein interaction (PPI) network of screened genes showing significantly causal effect on all the 5 sarcopenia-related traits; (D) the potential translational factors interacted with screened genes showing significantly causal effect on all the 5 sarcopenia-related traits; (E) the potential miRNA interacted with screened genes showing significantly causal effect on all the 5 sarcopenia-related traits; (F) the forest diagram showing 12 gene in B7 region with significantly causal effect on all the 5 sarcopenia-related traits.


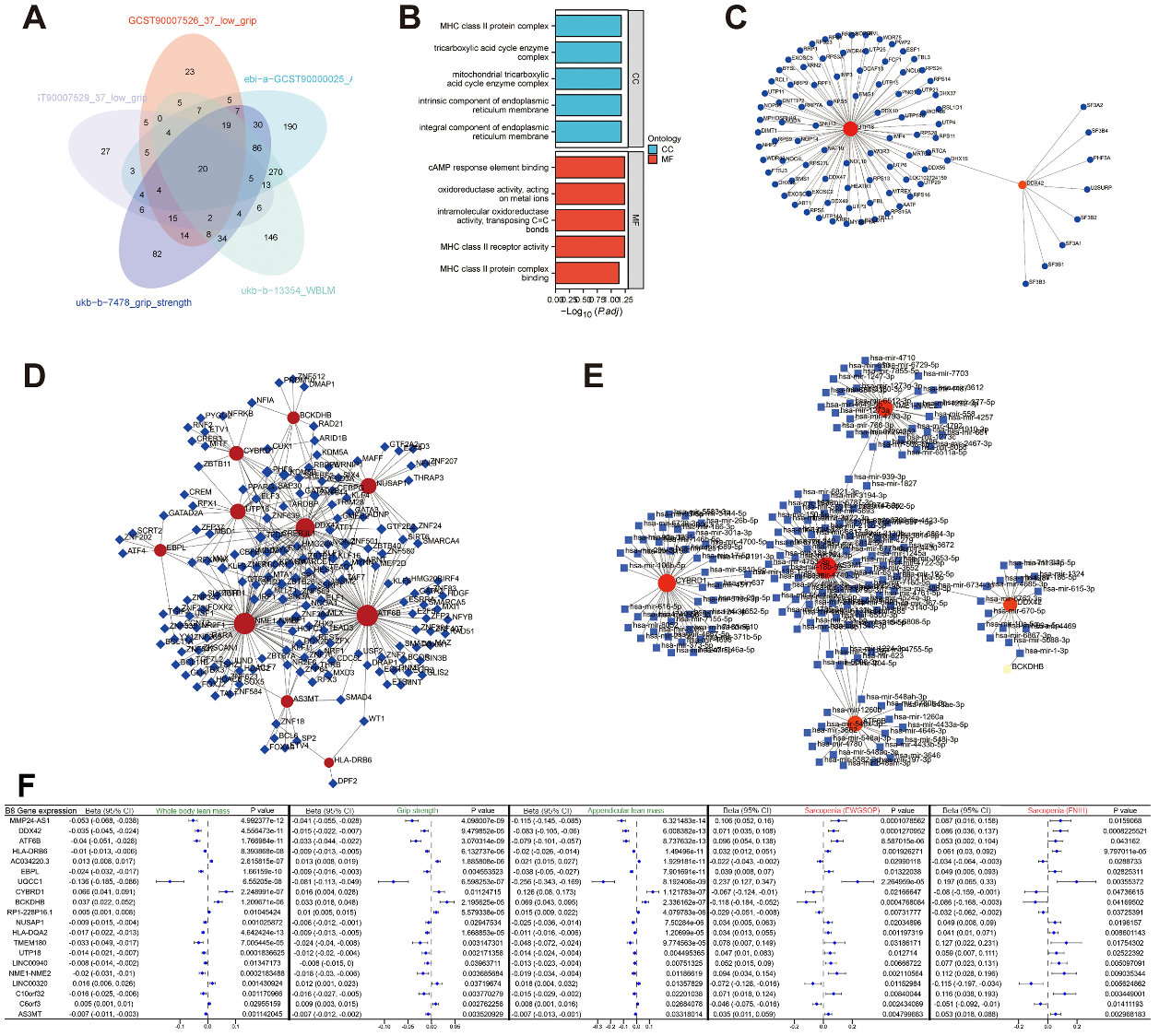


**Figure S8.** The causal effect of gene expression in [Brain Hippocampus](https://yanglab.westlake.edu.cn/data/SMR/GTEx_V8_cis_eqtl_summary/Brain_Hippocampus.zip) region (B8) on sarcopenia-related traits: (A) Wayne diagram of B8 region gene expression with significant causal effect on sarcopenia related traits; (B) the gene signaling enriched by the genes expressed in B8 region and showing significantly causal effect on all the 5 sarcopenia-related traits; (C) the protein-protein interaction (PPI) network of screened genes showing significantly causal effect on all the 5 sarcopenia-related traits; (D) the potential translational factors interacted with screened genes showing significantly causal effect on all the 5 sarcopenia-related traits; (E) the potential miRNA interacted with screened genes showing significantly causal effect on all the 5 sarcopenia-related traits; (F) the forest diagram showing 12 gene in B8 region with significantly causal effect on all the 5 sarcopenia-related traits.


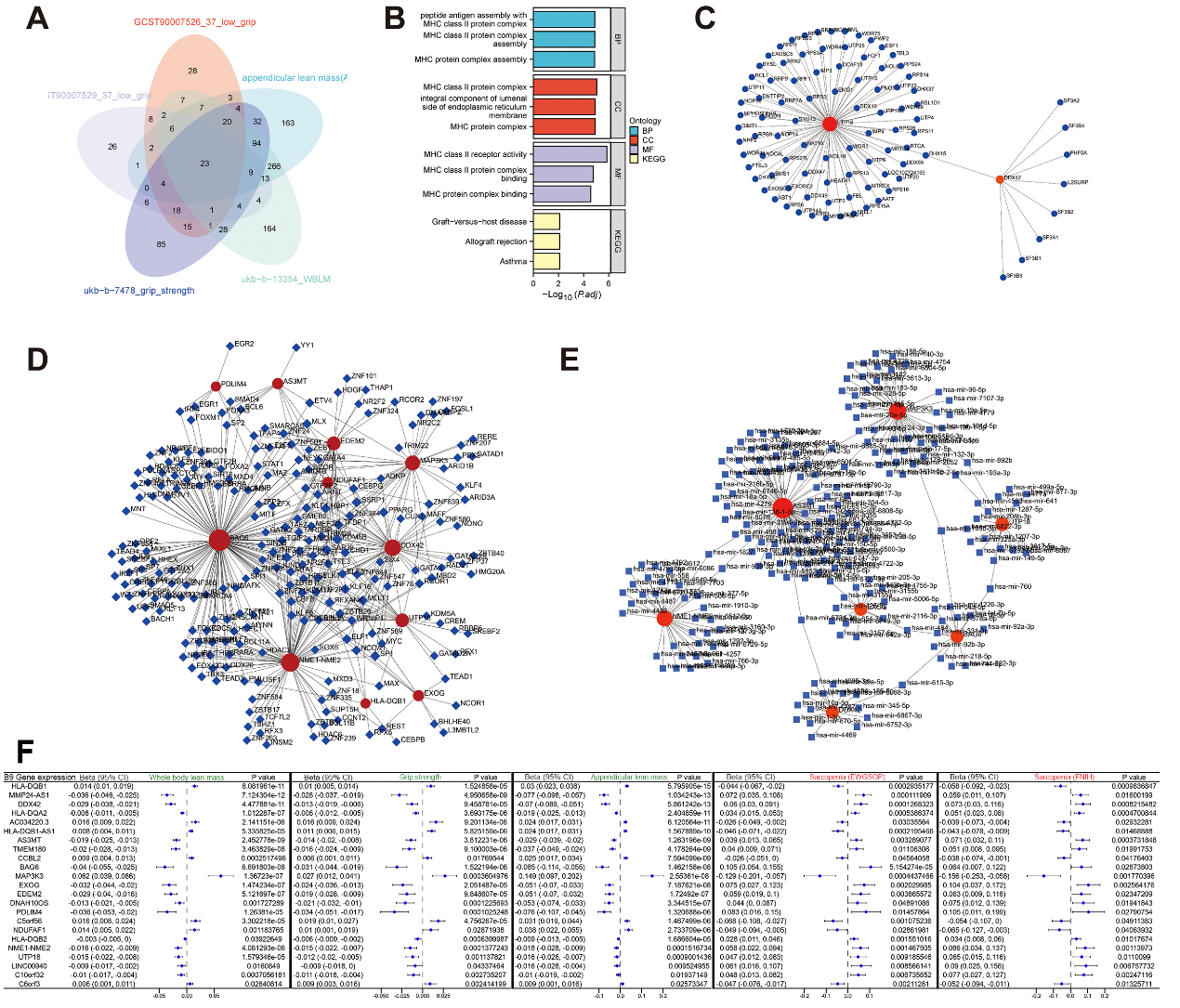


**Figure S9.** The causal effect of gene expression in Brain Hypothalamus region (B9) on sarcopenia-related traits: (A) Wayne diagram of B9 region gene expression with significant causal effect on sarcopenia related traits; (B) the gene signaling enriched by the genes expressed in B9 region and showing significantly causal effect on all the 5 sarcopenia-related traits; (C) the protein-protein interaction (PPI) network of screened genes showing significantly causal effect on all the 5 sarcopenia-related traits; (D) the potential translational factors interacted with screened genes showing significantly causal effect on all the 5 sarcopenia-related traits; (E) the potential miRNA interacted with screened genes showing significantly causal effect on all the 5 sarcopenia-related traits; (F) the forest diagram showing 12 gene in B9 region with significantly causal effect on all the 5 sarcopenia-related traits.


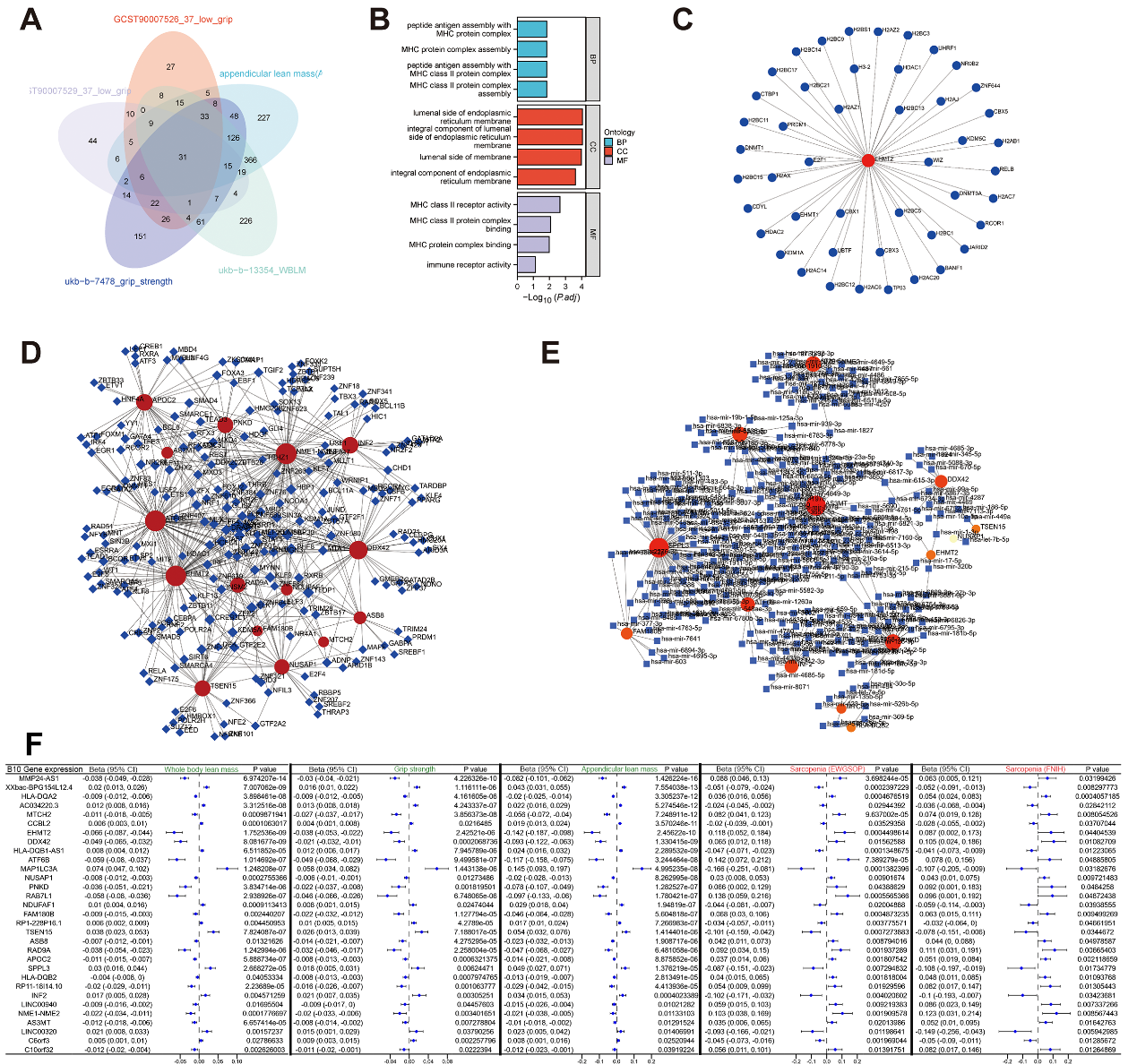


**Figure S10.** The causal effect of gene expression in [Brain Nucleus accumbens basal ganglia](https://yanglab.westlake.edu.cn/data/SMR/GTEx_V8_cis_eqtl_summary/Brain_Nucleus_accumbens_basal_ganglia.zip) (B10) on sarcopenia-related traits: (A) Wayne diagram of B10 region gene expression with significant causal effect on sarcopenia related traits; (B) the gene signaling enriched by the genes expressed in B10 region and showing significantly causal effect on all the 5 sarcopenia-related traits; (C) the protein-protein interaction (PPI) network of screened genes showing significantly causal effect on all the 5 sarcopenia-related traits; (D) the potential translational factors interacted with screened genes showing significantly causal effect on all the 5 sarcopenia-related traits; (E) the potential miRNA interacted with screened genes showing significantly causal effect on all the 5 sarcopenia-related traits; (F) the forest diagram showing 12 gene in B10 region with significantly causal effect on all the 5 sarcopenia-related traits.


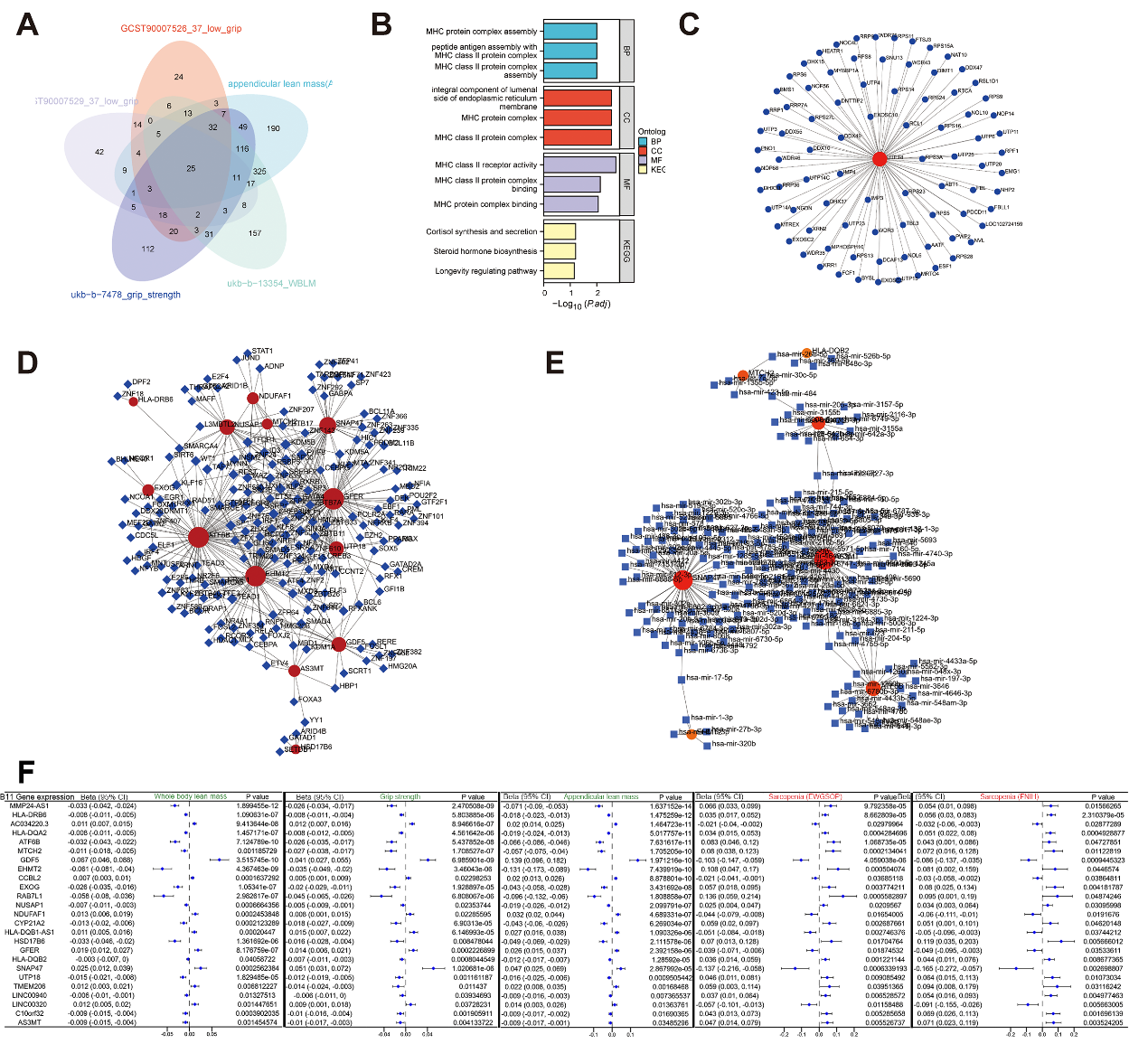


**Figure S11.** The causal effect of gene expression in [Brain Putamen basal ganglia](https://yanglab.westlake.edu.cn/data/SMR/GTEx_V8_cis_eqtl_summary/Brain_Putamen_basal_ganglia.zip) region (B11) on sarcopenia-related traits: (A) Wayne diagram of B11 region gene expression with significant causal effect on sarcopenia related traits; (B) the gene signaling enriched by the genes expressed in B11 region and showing significantly causal effect on all the 5 sarcopenia-related traits; (C) the protein-protein interaction (PPI) network of screened genes showing significantly causal effect on all the 5 sarcopenia-related traits; (D) the potential translational factors interacted with screened genes showing significantly causal effect on all the 5 sarcopenia-related traits; (E) the potential miRNA interacted with screened genes showing significantly causal effect on all the 5 sarcopenia-related traits; (F) the forest diagram showing 12 gene in B11 region with significantly causal effect on all the 5 sarcopenia-related traits.


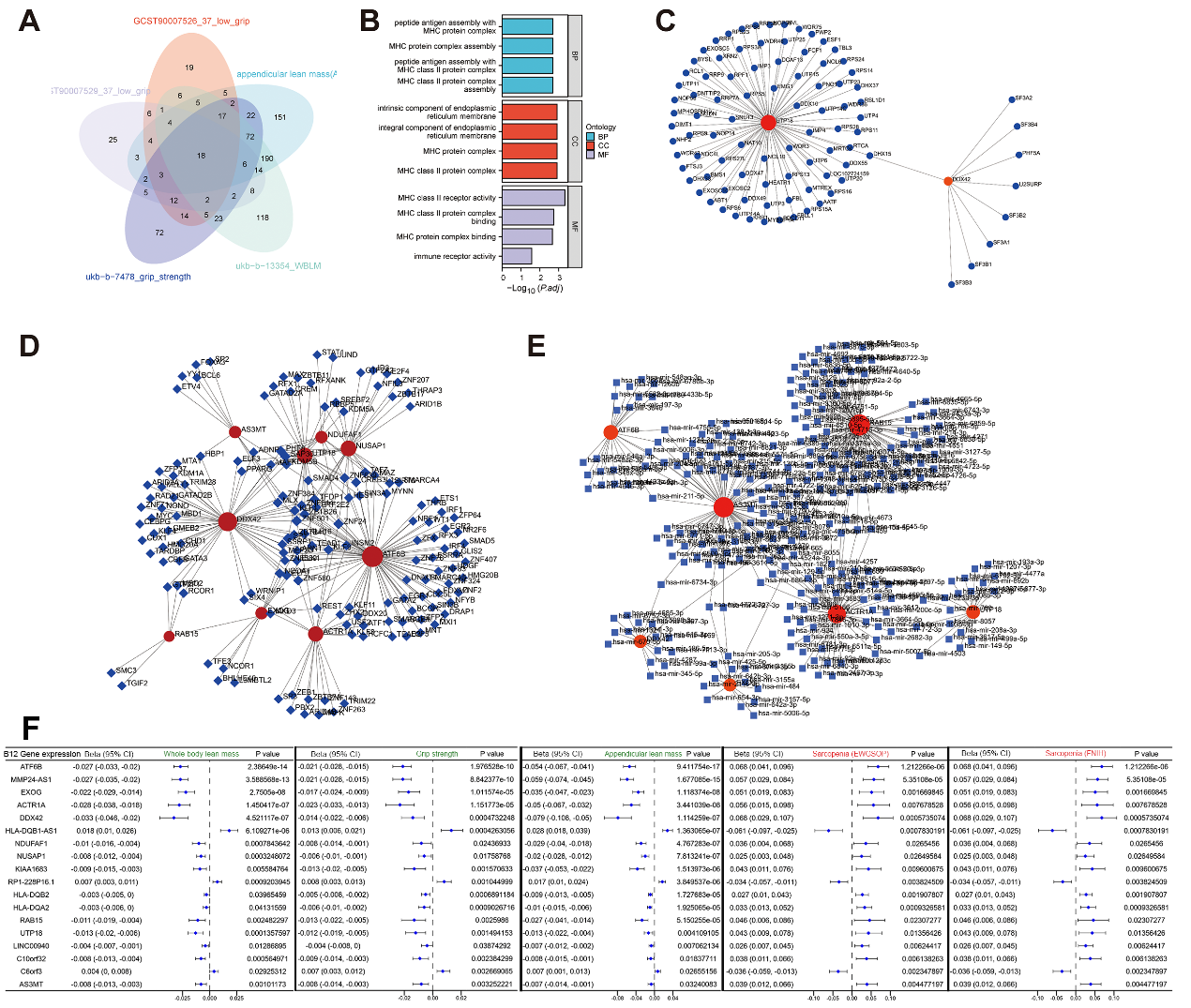


**Figure S12.** The causal effect of gene expression in [Brain Spinal cord cervical c-1](https://yanglab.westlake.edu.cn/data/SMR/GTEx_V8_cis_eqtl_summary/Brain_Spinal_cord_cervical_c-1.zip) region (B12) on sarcopenia-related traits: (A) Wayne diagram of B12 region gene expression with significant causal effect on sarcopenia related traits; (B) the gene signaling enriched by the genes expressed in B12 region and showing significantly causal effect on all the 5 sarcopenia-related traits; (C) the protein-protein interaction (PPI) network of screened genes showing significantly causal effect on all the 5 sarcopenia-related traits; (D) the potential translational factors interacted with screened genes showing significantly causal effect on all the 5 sarcopenia-related traits; (E) the potential miRNA interacted with screened genes showing significantly causal effect on all the 5 sarcopenia-related traits; (F) the forest diagram showing 12 gene in B12 region with significantly causal effect on all the 5 sarcopenia-related traits.


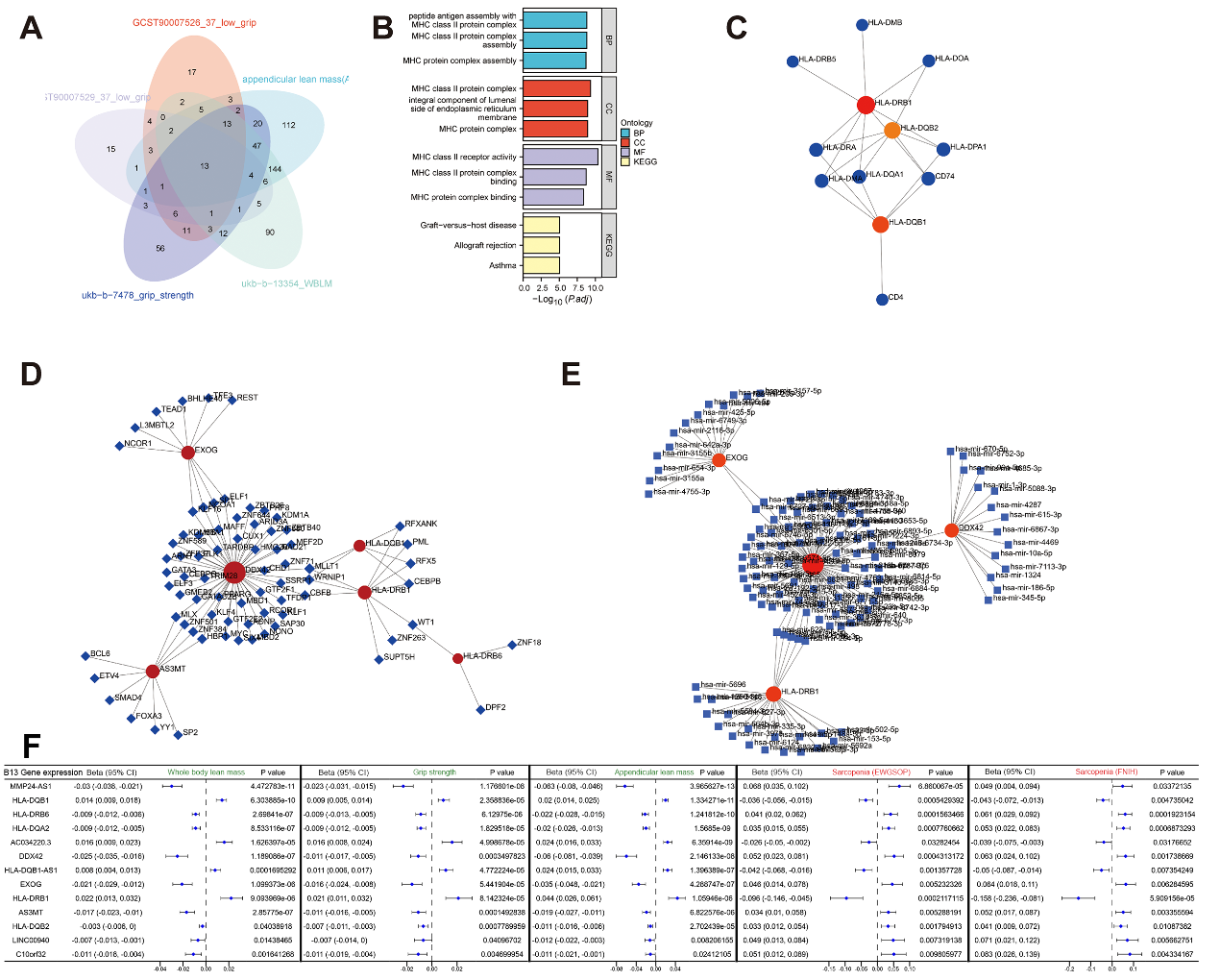


**Figure S13.** The causal effect of gene expression in [Brain Substantia nigra](https://yanglab.westlake.edu.cn/data/SMR/GTEx_V8_cis_eqtl_summary/Brain_Substantia_nigra.zip) region (B13) on sarcopenia-related traits: (A) Wayne diagram of B13 region gene expression with significant causal effect on sarcopenia related traits; (B) the gene signaling enriched by the genes expressed in B13 region and showing significantly causal effect on all the 5 sarcopenia-related traits; (C) the protein-protein interaction (PPI) network of screened genes showing significantly causal effect on all the 5 sarcopenia-related traits; (D) the potential translational factors interacted with screened genes showing significantly causal effect on all the 5 sarcopenia-related traits; (E) the potential miRNA interacted with screened genes showing significantly causal effect on all the 5 sarcopenia-related traits; (F) the forest diagram showing 12 gene in B13 region with significantly causal effect on all the 5 sarcopenia-related traits.
